# Global, regional, and national anemia burden among women of reproductive age (15–49 years) from 1990 to 2021: an analysis of the Global Burden of Disease Study 2021

**DOI:** 10.3389/fnut.2025.1588496

**Published:** 2025-07-28

**Authors:** Wenshuai Zheng, Bo Peng, Yamei Wu, Lixun Gauan, Shenyu Wang, Hongmei Ning

**Affiliations:** ^1^Department of Hematology, Hainan Hospital of Chinese PLA General Hospital, Sanya, Hainan, China; ^2^Senior Department of Hematology, The Fifth Medical Center of Chinese PLA General Hospital, Beijing, China

**Keywords:** anemia burden, women of reproductive age, Global Burden of Disease Study, epidemiology, projections

## Abstract

**Background:**

Anemia remains a significant global health challenge, disproportionately affecting women of reproductive age (WRA, 15 to 49 years) due to physiological and socioeconomic factors. However, there is a lack of high-quality data on anemia burden and causes analysis in this population. The aim of this study is to provide a comprehensive global assessment of anemia burden and its underlying causes among WRA.

**Methods:**

Using data from the Global Burden of Disease Study (GBD) 2021, we evaluated the prevalence, years lived with disability (YLDs), and underlying causes of anemia among WRA at global, regional, and national levels. We also evaluated the association between anemia burden and the socio-demographic index (SDI), quantified temporal trends of anemia burden from 1990 to 2021, and projected future burden to 2030.

**Results:**

Globally in 2021, there were 657.09 million (95% uncertainty interval [UI]: 643.59 to 671.22) anemia cases and 18.07 million (95% UI: 12.00 to 26.17) YLDs among WRA. The global age-standardized prevalence rates (ASPR) and YLDs rates per 100,000 were 33,716.77 (95% UI: 33,023.84 to 34,441.71) and 927.03 (95% UI: 615.40 to 1,342.99) in 2021, and they decreased by 0.181% and 0.539% annually from 1990 to 2021, respectively. However, the decline in ASPR stagnated after 2009, with a slight increase observed through 2021, primarily driven by rising mild anemia. An inverse relationship existed between SDI and anemia burden across regions and nations. The most common causes of anemia were dietary iron deficiency, hemoglobinopathies and hemolytic anemias, and other neglected tropical diseases, with HIV/AIDS and malaria being most prominent in specific regions. Projections indicate anemia cases and YLD counts will rise consistently from 2022 to 2030, while ASPR and age-standardized YLD rates will decline.

**Conclusion:**

Anemia among WRA is a major public health burden worldwide with persistent regional and socioeconomic disparities. Mitigating this burden requires continued efforts addressing underlying causes, reducing socioeconomic inequities, and improving access to healthcare and nutritional interventions.

## Introduction

Anemia represents a major global health challenge, particularly in developing countries (1). According to the Global Burden of Disease Study (GBD) in 2021, the global prevalence of anemia was 24.3%, corresponding to approximately 1.92 billion cases (2).

Women of reproductive age (WRA; 15–49 years) are among the highest-risk groups for anemia due to physiological factors such as menstruation, pregnancy, and peripartum blood loss (3, 4). In 2019, it is estimated that the anemia prevalence in non-pregnant and pregnant WRA was 30% and 36%, respectively, reflecting a modest decline since 2000 (5). Anemia impairs both cognitive and physical function, increases morbidity and mortality rates, and imposes substantial health and economic burdens (6). Maternal anemia is associated with stillbirth, low birth weight, infant mortality, and impaired cognitive development in infancy and early childhood (7, 8). Therefore, reducing anemia burden in this population carries critical health and socioeconomic implications.

Accurate, comprehensive assessments of anemia distribution and its determinants among WRA can offer valuable insights for stakeholders and guide effective intervention strategies (9). Previous studies have employed various data sources to evaluate anemia burden among WRA across low- and middle-income countries (LMICs) (1, 10). The GBD 2021 study provides detailed estimates of anemia prevalence and the years lived with disability (YLDs) across age groups and geographic regions (11). However, studies specifically quantifying the anemia burden in WRA using GBD data remain limited. One GBD 2019-based study accessed only prevalence of anemia, not burden, underlying causes, or the association with the socio-demographic index (SDI) (5).

Therefore, we conducted this study to evaluate the prevalence, YLDs and attributable causes of anemia in WRA at global, regional and national level, assess the association between anemia burden among WRA and SDI, and quantify and predict temporal trends of anemia burden among WRA. The findings would inform targeted interventions and prevention efforts.

## Materials and methods

### Data source

We performed a secondary analysis using data from the GBD 2021.^1^ Detailed GBD 2021 methodologies have been described extensively elsewhere (11). Briefly, the GBD 2021 integrates nationally representative surveys, census data, and results from meta-analyses, offering a thorough epidemiological evaluation of 371 diseases, injuries, impairments, along with 88 risk factors across 204 countries and territories using standardized methods. Various sources, such as peer-reviewed articles, surveys, disease registries, and hospital records, were utilized by the GBD 2021 to guarantee the comprehensiveness and quality of the analysis relating to anemia. These data were integrated using the following modeling tools: the Cause of Death Ensemble model (CODEm), spatiotemporal Gaussian process regression, and DisMod-MR 2.1 (a Bayesian meta-regression tool). The GBD study provides standardized estimates, including prevalence and YLDs, reported as absolute numbers, crude rates per 100,000 population, and age-standardized rates. All estimates are presented with 95% uncertainty intervals (UIs).

This research employed the SDI, a composite measure created from estimates of the total fertility rates for individuals under 25 years, the average years of education for those over 15 years, and income per capita adjusted for lag distribution, (12) to investigate the relationship between regional/national developmental status and the anemia burden among WRA. The SDI scale ranges from 0 (least developed) to 1 (most developed). In 2021, 204 countries and territories were categorized into five SDI quintiles: low, low-middle, middle, high-middle, and high.

This study adhered to the Guidelines for Accurate and Transparent Health Estimates Reporting (GATHER) (13). As no personal data were involved, ethical approval was not required.

### Disease definitions and causal attribution of anemia

Most surveys used a HemoCue test, adjusted for elevation, and excluded those with terminal or acute medical conditions. Published scientific literature studies and those from higher income locations typically measured Hb with a Coulter counter (2). As Hb concentration increases with altitude, the Hb concentration was adjusted for altitude using the formula recommended by WHO. The studies that reported altitude-adjusted Hb data were included directly, without any adjustment, while those that did not present altitude-adjusted Hb values, but did present altitude, were adjusted (2, 14). Anemia is defined by reduced concentrations of hemoglobin (Hb) in the blood, irrespective of etiology, red blood cell morphology, or function. Anemia diagnosis and severity classification (mild, moderate, or severe) follow WHO hemoglobin thresholds (g/L) (14).

Each anemia case was exclusively attributed to a single underlying cause. In GBD 2021, the following level 3 causes were included for anemia: chronic kidney disease; cirrhosis and other chronic liver diseases; dietary iron deficiency; endocrine, metabolic, blood and immune disorders; gynecological diseases; hemoglobinopathies and hemolytic anemias; HIV/AIDS; inflammatory bowel disease; intestinal nematode infections; malaria; maternal disorders; other neglected tropical diseases; other unspecified infectious diseases; schistosomiasis; upper digestive system diseases; and vitamin A deficiency. Vitamin A deficiency was excluded from further analysis due to zero recorded cases among WRA. Detailed cause definitions are provided elsewhere (2).

### Statistical analysis

We used joinpoint regression to evaluate temporal trends in anemia prevalence and YLDs among WRA, calculating the annual percentage change (APC) and average APC (AAPC) with 95% confidence intervals (CI). Positive APC or AAPC values indicate increasing trends, whereas negative values denote declines. To pinpoint years exhibiting the most significant trend changes, we analyzed temporal patterns in the dataset and identified the best-fitting model by dividing the logarithmic-scale data into multiple linear segments. The segmentation points, known as “joinpoints,” were determined through data-driven statistical methods. Further methodological details on joinpoint regression analysis are available in the literature (15).

To examine potential non-linear associations between anemia burden and SDI, we utilized Pearson correlation analysis to estimate the strength and direction of correlations between SDI, and age-standardized prevalence rates (ASPR) and age-standardized YLDs rates. This approach helped identify nations where anemia prevalence and YLDs substantially diverged from expectations based on socioeconomic status. Such comprehensive analysis clarifies how socioeconomic factors influence anemia prevalence and YLDs in WRA.

For forecasting anemia prevalence and YLDs (both counts and rates) among WRA from 2022 to 2030, we applied a Bayesian age-period-cohort (BAPC) model. In brief, the age-period-cohort model, a logarithmic linear Poisson model, postulates the multiplicative impact of age, period, and cohort, all following a Poisson distribution and utilizing a link function specific to the model (16).

All analyses and visualizations were conducted in R (version 4.3.3)^2^ and JD_GBDR (V2.37, Jingding Medical Technology Co., Ltd.). Statistical significance was defined as *p* < 0.05 (two-sided tests).

## Results

### Global level

**FIGURE 1:**
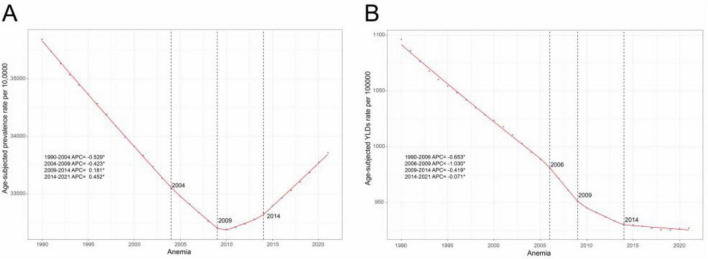
Joinpoint regression analysis of global anemia among WRA. **(A)** Age-standardized prevalence rates per 100,000 from 1990 to 2021, and **(B)** Age-standardized YLDs rates per 100,000 from 1990 to 2021. APC, annual percentage change; WRA, women of reproductive age; YLDs, years lost due to disability.

In 2021, anemia affected 657.09 million (95% UI: 643.59–671.22) WRA globally, accounting for 34.22% of all anemia cases worldwide. These cases resulted in 18.07 million (95% UI: 12.00–26.17) YLDs, representing 34.75% of global anemia-related YLDs (4). Compared to 1990, case numbers and YLDs in 2021 increased by 37.68% and 23.24%, respectively. However, the global ASPR and age-standardized YLDs rates decreased over the past 30 years. The ASPR per 100,000 decreased from 35,686.91 (95% UI: 35,020.87 to 36,352.73) in 1990 to 33,716.77 (95% UI: 33,023.84 to 34,441.71) in 2021, with an AAPC of −0.181 (95% CI: −0.186 to −0.177) (Table 1). Notably, the declining ASPR trend plateaued after 2009, with significant increases observed from 2009–2012 (APC = 0.181 [95% CI: 0.128–0.234]) and 2012–2021 (APC = 0.452 [95% CI: 0.434–0.471]). This reversal was primarily driven by rising mild anemia rates (2009–2012: APC = 0.764 [0.670–0.857]; 2012–2021: APC = 0.872 [0.832–0.911]) (Figure 1A and Supplementary Figure 1A). In the same period, the age-standardized YLDs rates per 100,000 declined from 1,096.13 (95% UI: 734.47 to 1,571.01) in 1990 to 927.03 (95% UI: 615.40 to 1,342.99) in 2021, with an AAPC of −0.539 (95% CI: −0.543 to −0.535) (Table 1 and Figure 1B). When considering different time period, 1990–2006 had the fastest downward trend (APC = −1.030 [95% CI: −1.171 to −0.889]), followed by 2006–2009 (APC = −0.653 [95% CI: −0.670 to −0.636]), 2009–2014 (APC = −0.419 [95% CI: −0.561 to −0.277]), and 2014–2021 (APC = −0.071 [95% CI: −0.120 to −0.022]) (Figure 1B). When considering the different severity of anemia among WRA, the ASPR and age-standardized YLDs rates in moderates and severe anemia showed consistently decreasing trends, while the trends of ASPR and age-standardized YLDs rates in mild anemia was similar to the trend of ASPR in all patients (Supplementary Figures 1A–F).

**TABLE 1:** The prevalence and YLDs of anemia among WRA, and their temporal trends from 1990 to 2021 at the global and regional levels.

Location	1990		2021		1990–2021, AAPC (%), (95% CI)	1990		2021		1990–2021, AAPC (%), (95% CI)
	Prevalence cases (95% UI)	ASPR per 100,000 (95% UI)	Prevalence cases (95% UI)	ASPR per 100,000 (95% UI)		YLDs cases (95% UI)	YLDs rates per 100,000 (95% UI)	YLDs cases (95% UI)	YLDs rates per 100,000 (95% UI)	
Global	477,263,149 (468,355,792 to 486,167,596)	35,686.91 (35,020.87 to 36,352.73)	657,091,159 (643,586,867 to 671,219,061)	33,716.77 (33,023.84 to 34,441.71)	−0.181 (−0.186 to −0.177)	14,659,274 (9,822,568 to 21,010,040)	1,096.13 (734.47 to 1,571.01)	18,066,561 (11,993,278 to 26,172,869)	927.03 (615.40 to 1,342.99)	−0.539 (−0.543 to −0.535)
High SDI	8,825,173 (6,872,262 to 11,529,928)	15,940.46 (14,538.71 to 17,634.68)	5,268,253 (3,993,360 to 7,026,430)	13,382.17 (12,261.13 to 14,696.72)	−0.565 (−0.579 to −0.551)	85,549 (45,226 to 143,679)	232.39 (144.22 to 357.51)	56,264 (31,915 to 98,626)	217.21 (134.15 to 326.92)	−0.213 (−0.226 to −0.201)
High-middle SDI	36,138,084 (32,960,231 to 39,978,993)	25,006.48 (24,038.22 to 26,134.58)	32,539,788 (29,813,886 to 35,736,216)	20,434.19 (19,321.20 to 21,576.76)	−0.647 (−0.653 to −0.642)	526,841 (326,955 to 810,499)	627.97 (408.19 to 936.27)	528,162 (326,206 to 794,941)	449.15 (293.83 to 660.84)	−1.071 (−1.079 to −1.065)
Middle SDI	155,162,373 (150,731,275 to 159,654,864)	34,706.99 (33,715.83 to 35,711.88)	186,516,523 (181,282,105 to 191,957,577)	30,157.88 (29,311.53 to 31,037.65)	−0.453 (−0.457 to −0.449)	4,399,436 (2,925,124 to 6,327,711)	984.07 (654.30 to 1,415.39)	4,887,188 (3,183,299 to 7,113,214)	790.21 (514.71 to 1,150.14)	−0.703 (−0.709 to −0.698)
Low-middle SDI	67,610,654 (66,035,082 to 69,256,763)	54,399.77 (52,667.57 to 56,033.62)	136,626,295 (131,629,496 to 142,098,946)	47,119.90 (45,373.92 to 49,107.46)	−0.463 (−0.466 to −0.46)	2,550,355 (1,729,395 to 3,651,308)	1.988.39 (1,333.63 to 2,832.08)	4,184,418 (2,778,256 to 5,995,915)	1,399.27 (922.55 to 2,010.79)	−1.13 (−1.134 to −1.126)
Low SDI	148,465,941 (143,738,488 to 152,924,983)	60,536.71 (59,125.99 to 62,010.59)	238,554,305 (229,714,883 to 248,616,729)	49,807.07 (47,985.49 to 51,802.12)	−0.633 (−0.64 to −0.627)	5,426,637 (3,639,685 to 7,729,206)	2,283.52 (1,548.45 to 3,269.28)	7,084,112 (4,670,609 to 10,180,034)	1,525.43 (1,012.81 to 2,185.81)	−1.291 (−1.298 to −1.283)
Andean Latin America	3,499,408 (2,970,127 to 4,152,159)	36,897.01 (31,316.39 to 43,779.49)	3,826,138 (3,458,797 to 4,335,071)	21,923.30 (19,818.48 to 24,839.42)	−1.648 (−1.68 to −1.609)	70,256 (43,746 to 10,6285)	740.76 (461.25 to 1,120.64)	72,405 (44,797 to 109,906)	414.87 (256.68 to 629.75)	−1.857 (−1.866 to −1.849)
Australasia	566,563 (489,877 to 660,931)	10,555.54 (9,126.82 to 12,313.70)	637,693 (536,697 to 761,850)	8,833.43 (7,434.43 to 10,553.28)	−0.571 (−0.579 to −0.566)	8,733 (4,861 to 14,040)	162.71 (90.57 to 261.58)	10,163 (6,091 to 15,950)	140.79 (84.38 to 220.95)	−0.466 (−0.473 to −0.459)
Caribbean	3,742,623 (3,476,606 to 4,057,407)	40,150.57 (37,296.76 to 43,527.54)	4,913,431 (4,510,220 to 5,374,841)	40,847.57 (37,495.49 to 44,683.48)	0.056 (0.047 to 0.066)	118,923 (77,799 to 172,753)	1,275.79 (834.62 to 1,853.28)	129,017 (83,314 to 184,721)	1,072.58 (692.63 to 1,535.67)	−0.564 (−0.572 to −0.558)
Central Asia	7,463,966 (7,069,243 to 7,950,346)	44,489.49 (42,136.71 to 47,388.59)	10,109,301 (9,306,925 to 11,126,289)	41,661.65 (38,354.96 to 45,852.78)	−0.213 (−0.216 to −0.21)	246,032 (163,813 to 355,827)	1,466.49 (976.42 to 2,120.93)	309,663 (203,631 to 453,727)	1,276.16 (839.19 to 1,869.86)	−0.451 (−0.458 to −0.445)
Central Europe	7,877,192 (7,339,541 to 8,476,878)	25,648.58 (23,897.96 to 27,601.20)	5,699,690 (5,235,716 to 6,300,205)	22,131.78 (20,330.17 to 24,463.56)	−0.472 (−0.477 to −0.467)	209,959 (136,915 to 310,621)	683.64 (445.80 to 1,011.40)	119,329 (76,656 to 176,221)	463.35 (297.65 to 684.26)	−1.247 (−1.255 to −1.24)
Central Latin America	7,436,849 (6,775,300 to 8,255,456)	17,743.42 (16,165.04 to 19,696.51)	9,662,216 (9,148,879 to 10,411,861)	14,168.60 (13,415.84 to 15,267.87)	−0.722 (−0.729 to −0.713)	149,842 (97,670 to 221,791)	357.51 (233.03 to 529.17)	212,922 (137,833 to 314,432)	312.23 (202.12 to 461.08)	−0.437 (−0.445 to −0.428)
Central Sub-Saharan Africa	8,470,371 (7,663,354 to 9,441,437)	68,522.45 (61,993.96 to 76,378.04)	15,562,051 (13,452,161 to 18,455,416)	47,659.20 (41,197.61 to 56,520.21)	−1.16 (−1.176 to −1.145)	257,681 (166,818 to 385,343)	2,084.56 (1,349.51 to 3,117.29)	388,384 (242,083 to 578,088)	1,189.44 (741.39 to 1,770.41)	−1.786 (−1.805 to −1.767)
East Asia	87,938,611 (84,441,241 to 91,697,982)	26,377.60 (25,328.55 to 27,505.24)	53,058,044 (50,845,485 to 55,529,904)	16,034.23 (15,365.59 to 16,781.23)	−1.598 (−1.612 to −1.588)	2,229,267 (1,462,274 to 3,275,007)	668.68 (438.62 to 982.35)	1,330,536 (866,601 to 1,954,024)	402.09 (261.89 to 590.51)	−1.633 (−1.642 to −1.626)
Eastern Europe	12,976,179 (11,564,976 to 14,814,914)	23,467.50 (20,915.33 to 26,792.86)	10,029,880 (8,923,692 to 11,518,772)	20,790.45 (18,497.48 to 23,876.70)	−0.391 (−0.402 to −0.38)	375,387 (241,659 to 558,681)	678.89 (437.04 to 1,010.38)	265,233 (178,504 to 398,345)	549.79 (370.01 to 825.71)	−0.68 (−0.692 to −0.665)
Eastern Sub-Saharan Africa	19,866,371 (19,270,937 to 20,499,550)	46,039.61 (44,659.71 to 47,506.98)	38,718,002 (36,854,366 to 41,056,125)	36,150.97 (34,410.89 to 38,334.07)	−0.776 (−0.79 to −0.768)	742,139 (500,573 to 1,068,115)	1,719.88 (1,160.06 to 2,475.32)	1,145,809 (772,686 to 1,641,497)	1,069.84 (721.46 to 1,532.66)	−1.524 (−1.543 to −1.503)
High-income Asia Pacific	9,287,675 (8,185,252 to 10,567,921)	19,294.16 (15,024.58 to 25,207.47)	10,027,106 (8,556,180 to 12,116,166)	13,849.72 (10,498.15 to 18,471.80)	−1.071 (−1.084 to −1.058)	121,191 (73,270 to 188,951)	187.03 (98.88 to 314.12)	169,194 (101,378 to 270,576)	147.91 (83.90 to 259.28)	−0.752 (−0.767 to −0.739)
High-income North America	69,456,475 (66,767,084 to 725,89,805)	12,488.92 (11,006.52 to 14,210.44)	62,350,437 (58,954,377 to 65,836,715)	11,933.89 (10,183.25 to 14,420.21)	−0.169 (−0.2 to −0.14)	1,744,217 (1,133,766 to 2,600,519)	162.96 (98.52 to 254.08)	1,370,484 (896,571 to 2,016,410)	201.37 (120.66 to 322.03)	0.698 (0.667 to 0.722)
North Africa and Middle East	32,899,572 (31,008,319 to 35,076,879)	42,111.48 (39,690.67 to 44,898.43)	52,598,989 (49,680,010 to 55,505,848)	33,009.39 (31,177.53 to 34,833.64)	−0.776 (−0.784 to −0.766)	828,932 (541,333 to 1,194,408)	1,061.03 (692.91 to 1,528.84)	1,352,713 (885,178 to 1,965,288)	848.92 (555.51 to 1,233.35)	−0.707 (−0.716 to −0.698)
Oceania	1,065,228 (923,796 to 1,287,491)	68,553.33 (59,451.43 to 82,857.24)	1,803,204 (1,460,740 to 2,209,501)	51,951.42 (42,084.80 to 63,657.08)	−0.916 (−0.938 to −0.893)	24,978 (14,620 to 38,133)	1,607.45 (940.86 to 2,454.04)	40,453 (24,975 to 63,879)	1,165.48 (719.54 to 1,840.39)	−1.03 (−1.051 to −1.012)
South Asia	158,269,885 (154,150,075 to 162,569,180)	62,092.84 (60,476.54 to 63,779.55)	269,609,286 (259,477,929 to 279,986,196)	54,561.63 (52,511.32 to 56,661.64)	−0.42 (−0.424 to −0.416)	6,309,143 (4,256,048 to 8,911,103)	2,475.22 (1,669.74 to 3,496.03)	8,175,476 (5,395,614 to 11,701,886)	1,654.50 (1,091.93 to 2,368.15)	−1.291 (−1.296 to −1.287)
Southeast Asia	46,157,759 (43,265,308 to 49,556,018)	38,404.46 (35,997.87 to 41,231.90)	59,303,618 (55,607,070 to 63,644,916)	32,370.64 (30,352.90 to 34,740.33)	−0.548 (−0.556 to −0.54)	1,166,603 (743,264 to 1,697,471)	970.64 (618.41 to 1,412.34)	1,363,292 (890,326 to 2,012,395)	744.15 (485.98 to 1,098.46)	−0.851 (−0.858 to −0.845)
Southern Latin America	1,823,634 (1,210,609 to 2,795,677)	14,715.14 (9,768.56 to 22,558.67)	2,469,894 (1,341,769 to 4,251,518)	14,170.51 (7,698.13 to 24,392.22)	−0.118 (−0.134 to −0.106)	19,573 (9,561 to 33,807)	157.94 (77.15 to 272.79)	24,558 (9,763 to 49,081)	140.90 (56.01 to 281.59)	−0.368 (−0.376 to −0.36)
Southern Sub-Saharan Africa	5,660,303 (4,996,603 to 6,406,334)	42,584.06 (37,590.86 to 48,196.66)	7,612,700 (7,022,151 to 8,319,444)	35,062.01 (32,342.11 to 38,317.08)	−0.621 (−0.627 to −0.613)	158,604 (100,819 to 238,186)	1,193.22 (758.49 to 1,791.94)	228,534 (147,659 to 332,235)	1,052.57 (680.08 to 1,530.18)	−0.403 (−0.412 to −0.393)
Tropical Latin America	16,345,975 (13,786,048 to 19,404,422)	40,976.57 (34,559.27 to 48,643.58)	20,687,060 (17,663,218 to 24,352,342)	34,129.81 (29,141.03 to 40,176.85)	−0.587 (−0.59 to −0.584)	476,734 (307,963 to 702,644)	1,195.09 (772.01 to 1,761.41)	528,536 (326,478 to 775,464)	871.99 (538.63 to 1,279.37)	−1.01 (−1.016 to −1.004)
Western Europe	10,742,563 (8,828,993 to 13,456,293)	11,243.04 (9,240.32 to 14,083.20)	8,009,089 (6,934,642 to 9,995,850)	8,595.32 (7,442.23 to 10,727.51)	−0.856 (−0.868 to −0.844)	125,202 (74,888 to 198,873)	131.04 (78.38 to 208.14)	99,616 (58,120 to 154,231)	106.91 (62.37 to 165.52)	−0.651 (−0.66 to −0.642)
Western Sub-Saharan Africa	26,347,248 (24,699,296 to 27,960,513)	60,422.74 (56,643.46 to 64,122.48)	67,485,513 (64,675,408 to 69,949,213)	56,289.56 (53,945.66 to 58,344.53)	−0.223 (−0.231 to −0.215)	934,545 (618,385 to 1,327,515)	2,143.21 (1,418.16 to 3,044.42)	2,044,463 (1,339,241 to 2,969,410)	1,705.28 (1,117.06 to 2,476.78)	−0.728 (−0.734 to −0.72)

AAPC, average annual percentage change; ASPR, age-standardized prevalence rates; CI, confidence interval; UI, uncertainty interval; WRA, women of reproductive age; YLDs, years lost due to disability.

### Regional level

In 2021, the highest ASPR per 100,000 among WRA were observed in Western Sub-Saharan Africa (56,289.56 [95% UI: 53,945.66 to 58,344.53]), South Asia (54,561.63 [95% UI: 52,511.32 to 56,661.64]), and Oceania (51,951.42 [95% UI: 42,084.80 to 63,657.08]). Western Europe (8,595.32 [95% UI: 7,442.23 to 10,727.51]), Australasia (8,833.43 [95% UI: 7,434.43 to 10,553.28]), and High-income North America (11,933.89 [95% UI: 10,183.25 to 14,420.21]) had the lowest ASPR per 100,000. From 1990 to 2021, ASPR declined in all GBD regions except the Caribbean (AAPC = 0.056 [0.047–0.066]). The most substantial reductions occurred in Andean Latin America (AAPC = −1.648 [−1.680 to −1.609]), East Asia (AAPC = −1.598 [−1.612 to −1.588]), and Central Sub-Saharan Africa (AAPC = −1.16 [−1.176 to −1.145]). Age-standardized YLD rates also declined universally except in the Caribbean (AAPC = 0.7 [0.67 to 0.72]), with regions exhibiting the greatest age-standardized YLD rates reductions similarly showing the largest ASRP decreases. Regions with the highest and lowest age-standardized YLDs were similar to regions with the highest and lowest ASPR (Table 1).

### National level

**FIGURE 2:**
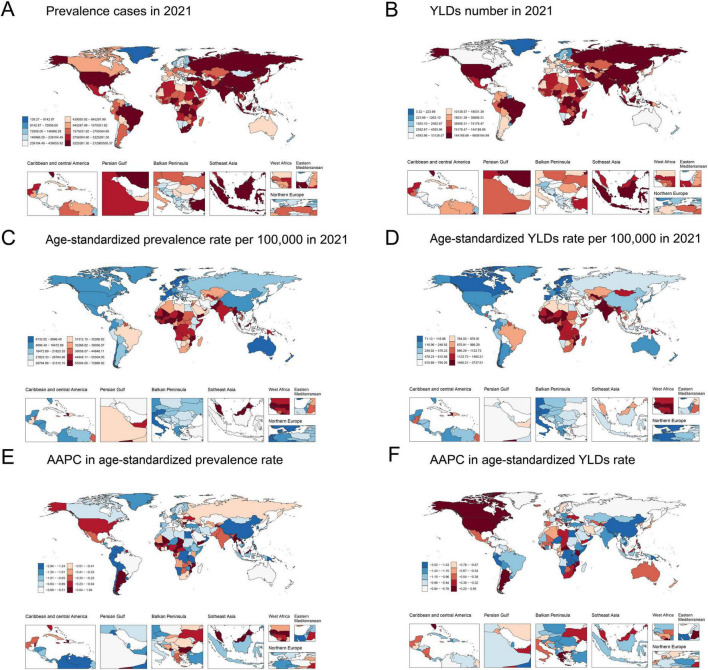
Global map of 2021 anemia among WRA. **(A)** Prevalence cases, **(B)** YLDs number, **(C)** Age-standardized prevalence rates per 100,000, and **(D)** Age-standardized YLDs rates per 100,000; AAPC in **(E)** Age-standardized prevalence rates and **(F)** Age-standardized YLDs rates from 1990 to 2021. AAPC, average annual percentage change; WRA, women of reproductive age; YLDs, years lost due to disability.

In 2021, the national ASPR per 100,000 of anemia among WRA ranged from 6,194.76 to 72,264.28. Liberia (72,264.28 [95% UI: 60,985.43 to 87,188.11]), Togo (68,744.88 [95% UI: 59,246.98 to 79,082.90]), and Senegal (67,940.94 [95% UI: 58,476.28 to 78,765.99]) had the highest ASPR in 2021, while Norway (6,194.76 [95% UI: 4,952.15 to 7,953.54]), Monaco (6,296.68 [95% UI: 5,041.68 to 7,854.56]), and Iceland (6,578.64 [95% UI: 5,454.72 to 8,001.69]) had the lowest ASPR. The national age-standardized YLDs rates per 100,000 of anemia among WRA varied from 71.85 to 3,700.01 in 2021, with the highest age-standardized YLD rates in Mozambique (3,700.01 [95% UI: 2,619.61 to 5,125.53]), Liberia (3,392.90 [95% UI: 2,178.87 to 4,885.07]), and Yemen (3,058.19 [95% UI: 2,055.13 to 4,242.27]), and the lowest rates in Monaco (71.85 [95% UI: 37.14 to 114.57]), United Kingdom (84.67 [95% UI: 47.39 to 136.02]), and Germany (87.24 [95% UI: 30.64 to 198.20]). India (209.97 million [95% UI: 202.25 to 217.61]), China (50.34 million [95% UI: 48.11 to 52.73]) and Pakistan (34.32 million [95% UI: 30.29 to 38.99]) had the highest prevalence cases and YLD counts (Supplementary Table 1 and Figures 2A–D).

The ASPR and age-standardized YLDs rates of anemia among WRA decreased in most countries from 1990 to 2021. The steepest ASPR reductions were observed in Namibia (AAPC = −2.91 [95% CI: −2.94 to −2.88]), Singapore (AAPC = −2.40 [95% CI: −2.42 to −2.38]), and Oman (AAPC = −2.19 [95% CI: −2.21 to −2.17]). The largest decreases in age-standardized YLD rates were found in Malawi (AAPC = −3.49 [95% CI: −3.54 to −3.44]), United Republic of Tanzania (AAPC = −3.41 [95% CI: −3.44 to −3.38]), and Singapore (AAPC = −2.74 [95% CI: −2.77 to −2.73]) (Supplementary Table 1 and Figures 2E–F).

### Association with the socio-demographic index

**FIGURE 3:**
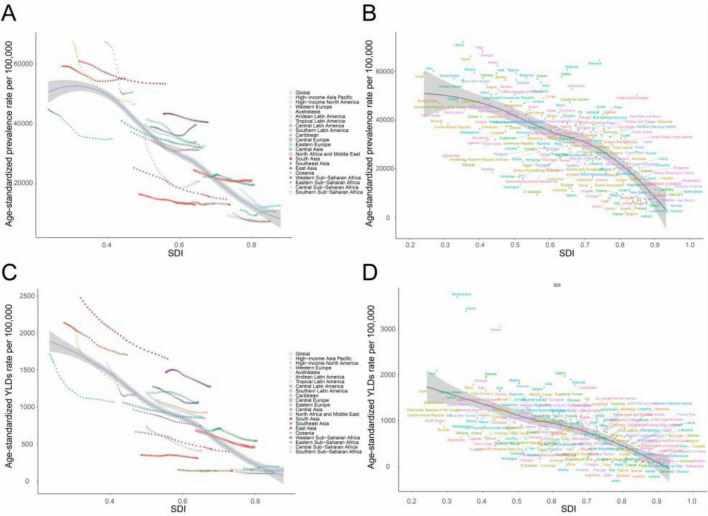
Correlation between SDI and anemia burden among WRA by 21 GBD regions and 204 national. **(A)** Trends in age-standardized prevalence rates per 100,000 across 21 GBD regions, correlated with SDI from 1990 to 2021, **(B)** Age-standardized prevalence rates per 100,000 at the national level in relation to SDI in 2021. **(C)** Trends in age-standardized YLD rates per 100,000 across 21 GBD regions, correlated with SDI from 1990 to 2021, and **(D)** Age-standardized YLDs rates per 100,000 at the national level in relation to SDI in 2021. GBD, Global Burden of Disease Study; SDI, socio-demographic index; WRA, women of reproductive age; YLDs, years lost due to disability.

In 2021, the low-SDI regions had the highest ASPR per 100,000 (49,807.07 [95% UI: 47,985.49 to 51,802.12]) and age-standardized YLDs rates per 100,000 (1,525.43 [95% UI: 1,012.81 to 2,185.81]). Both metrics decreased incrementally with rising SDI (Table 1). Regionally and nationally, higher SDI correlated strongly with lower ASPR and age-standardized YLD rates from 1990 to 2021 (Table 1 and Figures 3A–D; Supplementary Table 1), indicating lower anemia burden in regions with higher socioeconomic development.

### Underlying causes of anemia

**FIGURE 4:**
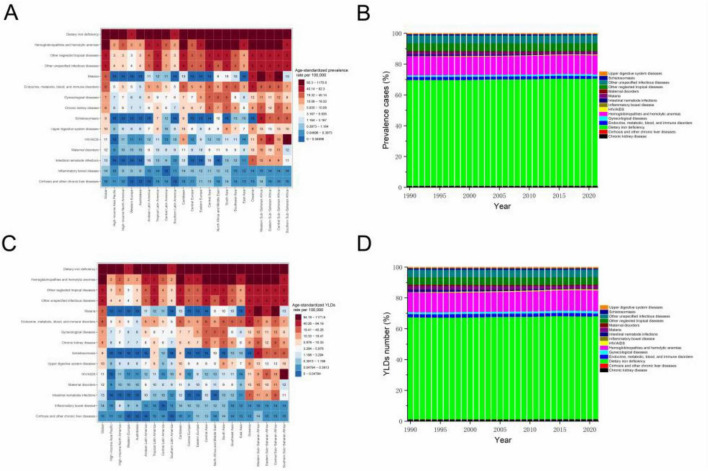
**(A)** Ranking of all underlying causes of anemia among WRA according to the age-standardized prevalence rates per 100,000 in 2021, stratified by global and regions, **(B)** The proportion of prevalence cases of anemia among WRA due to each cause from 1990 to 2021, **(C)** Ranking of all underlying causes of anemia among WRA according to the age-standardized YLDs rates per 100,000 in 2021, stratified by global and regions, and **(D)** The proportion of YLDs number of anemia among WRA due to each cause from 1990 to 2021. Abbreviations as in Figure 1.

Globally, the leading cause of anemia among WRA in 2021 were dietary iron deficiency, hemoglobinopathies and hemolytic anemias, and other neglected tropical diseases. Dietary iron deficiency accounted for a substantial proportion of anemia cases (455.46 million; 95% UI: 445.04–466.17) in 2021, representing 69.31% of total prevalence. However, its contribution to YLDs was slightly lower at 66.46% with 12.01 million (95% UI: 7.89–17.44), suggesting that dietary iron deficiency-related anemia tends to be less severe. Geographic variations existed: HIV/AIDS was the second-leading cause in Southern Sub-Saharan Africa, while malaria ranked third in Central, Eastern, and Western Sub-Saharan Africa. Etiologic proportions remained stable from 1990 to 2021 (Figures 4A–D).

### Predictions of anemia burden

**FIGURE 5:**
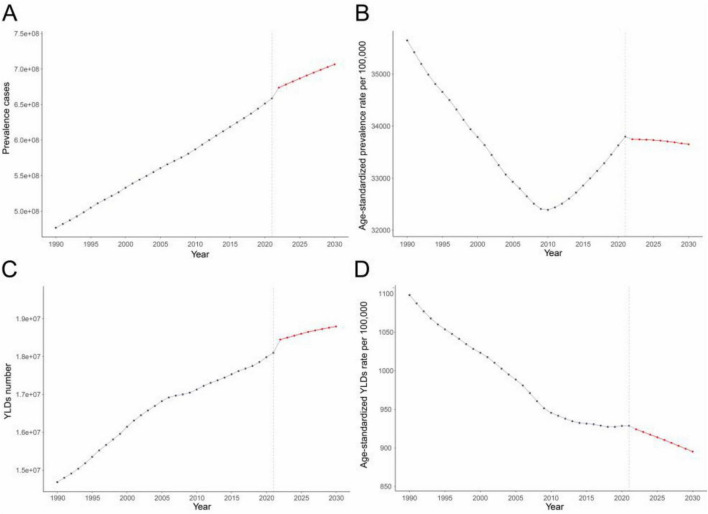
**(A)** Prevalence cases, **(B)** Age-standardized prevalence rates per 100,000, **(C)** YLDs number, and **(D)** Age-standardized YLDs rates per 100,000 of anemia among WRA from 1990 to 2021, and their predictions to 2030. The known data from 1990 to 2021 and predicted data from 2022 to 2030 were divided by a gray dashed line. Abbreviations as in Figure 1.

BAPC analysis projected consistent increases in anemia cases and YLDs among WRA from 2022 to 2030. Globally, cases will rise to 706.55 million (95% UI: 673.05–740.04) and YLDs to 18.75 million (95% UI: 17.98–19.61) by 2030, with an increase of 7.3% and 3.8%, respectively, compared with those in 2021. Conversely, ASPR (33,648.76 [95% UI: 32,053.67–35,243.86]) and YLD rates (895.09 [95% UI: 856.14–934.04]) per 100,000 will decline by 0.4% and 3.6%, respectively (Supplementary Table 2 and Figure 5). Trends for moderate/severe anemia align with overall patterns, but the ASPR and age-standardized YLDs rates of mild anemia showed a rising trend (Supplementary Tables 3–5 and Supplementary Figures 2–4).

## Discussion

This study presents the first systematic assessment of anemia burden and its attributable causes among WRA globally, regionally, and nationally from 1990 to 2021, using data from GBD 2021 study. Despite worldwide reductions in ASPR and YLD rates over the past 30 years, anemia a critical health challenge for WRA, especially in LMICs. Our analysis highlights persistent inequalities in anemia burden stratified by socioeconomic development, with the highest prevalence concentrated in low-SDI settings. In addition, we quantify and predict temporal trends of this burden.

From 1990 to 2021, the global number of anemia cases among WRA increased by 37.68%, reaching 657.09 million in 2021. Concurrently, anemia-attributable YLDs rose by 23.24% to 18.07 million in 2021. These increases in absolute prevalence and YLDs are primarily attributable to demographic shifts, including population growth, aging, and extended survival among individuals with chronic anemia-inducing conditions. In contrast, ASPR declined by 0.18% annually, and the age-standardized YLD rates decreased by 0.54% per year during this timeframe. These reductions likely reflect advancements in healthcare infrastructure, such as improved screening, preventive measures, and therapeutic interventions. These findings align with prior research documenting global declines in anemia rates, especially in high-income nations, driven by improved healthcare access, nutritional programs, and public health initiatives (2, 17, 18). Notably, the decline in ASPR stagnated after 2009, with a slight increase observed through 2021. Thus trend was primarily driven by rising ASPR in mild anemia, which may partially reflect successful management converting moderate/severe cases to mild anemia-evidenced by persistently declining ASPR for moderate/severe anemia. This stagnation underscores enduring challenges, particularly in mild anemia, which is frequently overlooked despite its population-wide impact.

We observed a consistent inverse correlation between SDI and anemia burden, with lower SDI areas exhibiting elevated ASPR and YLDs rates. Low SDI reflects key socio-demographic challenges, such as poor nutrition, limited healthcare access, low educational attainment, and gender inequity (17). Micronutrient deficiencies, particularly iron insufficiency, are prevalent among WRA in LMICs, heightening anemia risk (19). Furthermore, women without formal education face an 8% greater likelihood of anemia compared to those with secondary or higher education (20). Gender disparities in household roles, such as food allocation and labor division, likely widen gaps in anemia prevalence, as women are disproportionately impacted by food insecurity, (21, 22) insufficient access to iron-rich diets, (23) and limited healthcare access (24). Collectively, these factors elucidate the observed negative association between SDI and anemia burden and underscore the importance of addressing socioeconomic and gender disparities in anemia prevention strategies.

Dietary iron deficiency persists as the dominant contributor to anemia among WRA worldwide, accounting for 69.31% of global anemia cases in 2021. This aligns with prior evidence identifying iron-poor diets as a key driver of anemia, particularly in LMICs (1, 2, 10, 17). Consequently, iron supplementation remains central to public health strategies, including fortified food programs, (25) and oral/intravenous iron therapies (26). Improvements in treating Hb disorders have allowed individuals who would have previously died in childhood to reach adulthood. This elevates risks of complications like anemia (27). Alongside disorder-specific care, enhancing public awareness of genetic risks, carrier screening, and genetic counseling for at-risk couples is critical (28).

We identified pronounced regional heterogeneity in the causes of anemia among WRA. For instance, HIV/AIDS was as the second-leading cause of anemia in Southern Sub-Saharan Africa, while malaria ranked third in malaria endemic zones, particularly across Central, Eastern, and Western Sub-Saharan Africa. Therefore, effective prevention of anemia requires region-specific interventions. For example, antiretroviral therapy is critical to reduce HIV/AIDS-related anemia severity (29), while malaria control through insecticide-treated bed nets, vector management, vaccine, and antimalarial drug access can mitigate malaria-related anemia (30).

Our predictive analysis indicates that absolute prevalence and YLDs will continue rising due to the population growth and aging, while ASPR and age-standardized YLDs rates are projected to decline. The ASPR decrease will be less pronounced than that of YLD rates because rising mild anemia ASPR attenuates the overall ASPR decline. Conversely, mild anemia contributes minimally to total YLDs, so its increase has limited impact on age-standardized YLD rates.

In 2012, the World Health Assembly’s Nutrition Goals aimed to half anemia prevalence among WRA by 2025 (vs. a 1993–2005 baseline of 30.2%) (31). However, WHO reported that the prevalence rates rose from 28⋅5% in 2013 to 29⋅9% in 2019 after a slight decrease between 2000 and 2011, (5) prompting an extension of the target to 2030 (32). Our predictions using the BAPC model indicate that the ASPR of anemia among WRA will be 33,648.76 per 100,000 by 2030, falls far short of the target of the WHO’s 50% reduction goal. This underscores the urgent need for intensified efforts, especially in low-SDI regions.

These findings inform targeted public health strategies to mitigate anemia burden among WRA. First, there is a need for comprehensive strategies that address the underlying causes of anemia, including dietary iron deficiency, hemoglobinopathies, and infectious diseases such as malaria and HIV/AIDS. Second, efforts to improve access to healthcare and nutrition in low-SDI regions are critical. This includes increasing the availability of iron supplements, particularly for pregnant women, and improving the coverage of antenatal care services. Third, the stagnation in the decline of anemia prevalence since 2009 highlights the need for more effective and sustainable interventions. This may include integrating anemia prevention and control into broader maternal and child health programs, as well as leveraging new technologies and innovations to improve the delivery of interventions.

The study has several limitations. First, our study depends on data quality and availability, which have notable disparities across regions. In particular, data from low-SDI regions may be less reliable due to limited healthcare infrastructure. Second, the GBD study relies on modeling techniques to estimate anemia prevalence and YLDs, which may introduce inaccuracies. Third, the GBD 2021 study assigns one primary cause per anemia case, potentially underestimating multifactorial etiologies.

In summary, anemia among WRA remains a significant global health challenge with marked geographical heterogeneity. Despite declines in ASPR and YLDs rates from 1990 to 2021, it disproportionately affects LMICs. Achieving the WHO Global Nutrition Targets demands integrated approaches, including addressing etiology, improving access to healthcare and nutrition, and reducing socioeconomic inequities. While such comprehensive interventions may exceed current capacities for many nations, their implementation is critical to mitigating this preventable burden. Prioritizing anemia control will improve health for millions of women and advance global health equity.

## Data Availability

The original contributions presented in this study are included in this article/Supplementary material, further inquiries can be directed to the corresponding authors.

## Footnotes

1
https://vizhub.healthdata.org/gbd-results/

2
https://www.r-project.org

## References

[1] Kinyoki D Osgood-Zimmerman A Bhattacharjee N Kassebaum N Hay S. Anemia prevalence in women of reproductive age in low– and middle-income countries between 2000 and 2018. *Nat Med.* (2021) 27:1761–82. 10.1038/s41591-021-01498-0 34642490 PMC8516651

[2] GBD 2021 Anaemia Collaborators. Prevalence, years lived with disability and trends in anaemia burden by severity and cause, 1990-2021: findings from the global burden of disease study 2021. *Lancet Haematol.* (2023) 10:e713–34. 10.1016/S2352-302600160-6 37536353 PMC10465717

[3] Chaparro C Suchdev P. Anemia epidemiology, pathophysiology, and etiology in low- and middle-income countries. *Ann N Y Acad Sci.* (2019) 1450:15–31. 10.1111/nyas.14092 31008520 PMC6697587

[4] Percy L Mansour D Fraser I. Iron deficiency and iron deficiency anaemia in women. *Best Pract Res Clin Obstet Gynaecol.* (2017) 40:55–67. 10.1016/j.bpobgyn.2016.09.007 28029503

[5] Stevens G Paciorek C Flores-Urrutia M Borghi E Namaste S Wirth J National, regional, and global estimates of anaemia by severity in women and children for 2000-19: a pooled analysis of population-representative data. *Lancet Glob Health.* (2022) 10:e627–39. 10.1016/S2214-109X00084-5 35427520 PMC9023869

[6] Martinsson A Andersson C Andell P Koul S Engström G Smith J. Anemia in the general population: prevalence, clinical correlates and prognostic impact. *Eur J Epidemiol.* (2014) 29:489–98. 10.1007/s10654-014-9929-9 24952166

[7] Smith E Shankar A Wu L Aboud S Adu-Afarwuah S Ali H Modifiers of the effect of maternal multiple micronutrient supplementation on stillbirth, birth outcomes, and infant mortality: a meta-analysis of individual patient data from 17 randomised trials in low-income and middle-income countries. *Lancet Glob Health.* (2017) 5:e1090–100. 10.1016/S2214-109X30371-6 29025632

[8] Young M Oaks B Tandon S Martorell R Dewey K Wendt A. Maternal hemoglobin concentrations across pregnancy and maternal and child health: a systematic review and meta-analysis. *Ann N Y Acad Sci.* (2019) 1450:47–68. 10.1111/nyas.14093 30994929 PMC6767572

[9] Williams A Addo O Grosse S Kassebaum N Rankin Z Ballesteros K Data needed to respond appropriately to anemia when it is a public health problem. *Ann N Y Acad Sci.* (2019) 1450:268–80. 10.1111/nyas.14175 31267542 PMC8291089

[10] Alem A Efendi F McKenna L Felipe-Dimog E Chilot D Tonapa S Prevalence and factors associated with anemia in women of reproductive age across low- and middle-income countries based on national data. *Sci Rep.* (2023) 13:20335. 10.1038/s41598-023-46739-z 37990069 PMC10663544

[11] GBD 2021 Diseases and Injuries Collaborators. Global incidence, prevalence, years lived with disability (YLDs), disability-adjusted life-years (DALYs), and healthy life expectancy (HALE) for 371 diseases and injuries in 204 countries and territories and 811 subnational locations, 1990-2021: a systematic analysis for the global burden of disease study 2021. *Lancet.* (2024) 403:2133–61. 10.1016/S0140-673600757-8 38642570 PMC11122111

[12] GBD 2019 Demographics Collaborators. Global age-sex-specific fertility, mortality, healthy life expectancy (HALE), and population estimates in 204 countries and territories, 1950-2019: a comprehensive demographic analysis for the global burden of disease study 2019. *Lancet.* (2020) 396:1160–203. 10.1016/S0140-673630977-6 33069325 PMC7566045

[13] Stevens G Alkema L Black R Boerma J Collins G Ezzati M Guidelines for accurate and transparent health estimates reporting: the GATHER statement. *Lancet.* (2016) 388:e19–23. 10.1016/S0140-673630388-9 27371184

[14] World Health Organization. *Haemoglobin Concentrations for the Diagnosis of Anemia and Assessment of Severity.* Geneva: World Health Organization (2011).

[15] Yang C Jia Y Zhang C Jin Z Ma Y Bi X Global, regional, and national burdens of heart failure in adolescents and young adults aged 10-24 years from 1990 to 2021: an analysis of data from the global burden of disease study 2021. *EClinicalMedicine.* (2025) 79:102998. 10.1016/j.eclinm.2024.102998 39737218 PMC11683260

[16] Jürgens V Ess S Cerny T Vounatsou PA. Bayesian generalized age-period-cohort power model for cancer projections. *Stat Med.* (2014) 33:4627–36. 10.1002/sim.6248 24996118

[17] Safiri S Kolahi A Noori M Nejadghaderi S Karamzad N Bragazzi N Burden of anemia and its underlying causes in 204 countries and territories, 1990-2019: results from the global burden of disease study 2019. *J Hematol Oncol.* (2021) 14:185. 10.1186/s13045-021-01202-2 34736513 PMC8567696

[18] Kassebaum N Jasrasaria R Naghavi M Wulf S Johns N Lozano R A systematic analysis of global anemia burden from 1990 to 2010. *Blood.* (2014) 123:615–24. 10.1182/blood-2013-06-508325 24297872 PMC3907750

[19] Torheim L Ferguson E Penrose K Arimond M. Women in resource-poor settings are at risk of inadequate intakes of multiple micronutrients. *J Nutr.* (2010) 140:2051S–8S. 10.3945/jn.110.123463 20881075

[20] Balarajan Y Ramakrishnan U Ozaltin E Shankar A Subramanian S. Anaemia in low-income and middle-income countries. *Lancet.* (2011) 378:2123–35. 10.1016/S0140-673662304-5 21813172

[21] Quisumbing A Kumar N Behrman J. Do shocks affect men’s and women’s assets differently? Evidence from Bangladesh and Uganda. *Dev Policy Rev.* (2017) 36:3–34. 10.1111/dpr.12235

[22] Santos M Brewer J Lopez M Paz-Soldan V Chaparro M. Determinants of food insecurity among households with children in Villa el Salvador, Lima, Peru: the role of gender and employment, a cross-sectional study. *BMC Public Health.* (2022) 22:717. 10.1186/s12889-022-12889-4 35410187 PMC8996213

[23] Girard A Self J McAuliffe C Olude O. The effects of household food production strategies on the health and nutrition outcomes of women and young children: a systematic review. *Paediatr Perinat Epidemiol.* (2012) 26:205–22. 10.1111/j.1365-3016.2012.01282.x 22742612

[24] Yang F Liu X Zha P. Trends in socioeconomic inequalities and prevalence of anemia among children and nonpregnant women in low- and middle-income countries. *JAMA Netw Open.* (2018) 1:e182899. 10.1001/jamanetworkopen.2018.2899 30646183 PMC6324516

[25] Keats E Neufeld L Garrett G Mbuya M Bhutta Z. Improved micronutrient status and health outcomes in low- and middle-income countries following large-scale fortification: evidence from a systematic review and meta-analysis. *Am J Clin Nutr.* (2019) 109:1696–708. 10.1093/ajcn/nqz023 30997493 PMC6537942

[26] Christian P. Anemia in women – an intractable problem that requires innovative solutions. *Nat Med.* (2021) 27:1675–7. 10.1038/s41591-021-01514-3 34642492

[27] Weatherall D. The challenge of haemoglobinopathies in resource-poor countries. *Br J Haematol.* (2011) 154:736–44. 10.1111/j.1365-2141.2011.08742.x 21726207

[28] Hoppe C. Prenatal and newborn screening for hemoglobinopathies. *Int J Lab Hematol.* (2013) 35:297–305. 10.1111/ijlh.12076 23590658

[29] Berhane K Karim R Cohen M Masri-Lavine L Young M Anastos K Impact of highly active antiretroviral therapy on anemia and relationship between anemia and survival in a large cohort of HIV-infected women: women’s Interagency HIV Study. *J Acquir Immune Defic Syndr.* (2004) 37:1245–52. 10.1097/01.qai.0000134759.01684.27 15385731

[30] The Lancet. Malaria vaccine approval: a step change for global health. *Lancet.* (2021) 398:1381. 10.1016/S0140-673602235-2 34656212

[31] World Health Organization. *Global Nutrition Targets 2025: Anemia Policy Brief.* Geneva: World Health Organization (2014).

[32] World Health Organization. *WHO/UNICEF Discussion Paper. The Extension of the 2025 Maternal, Infant and Young Child Nutrition Targets to 2030.* Geneva: World Health Organization (2025).

