# Supplementary material for: Global, regional, and national anemia burden among women of reproductive age (15–49 years) from 1990 to 2021: an analysis of the Global Burden of Disease Study 2021
Source: Front Nutr. 2025 Jul 28;12:1588496. doi: 10.3389/fnut.2025.1588496 (PMC12338041; doi:10.3389/fnut.2025.1588496)
Supplement: Supplementary file 1 [file Table_1.docx]

Supplementary Table 1 The prevalence and YLDs of anemia among WRA, and their temporal trends from 1990 to 2021 at the national levels.

| Location | 1990 | | 2021 | | 1990–2021, AAPC (%),  (95% CI) | 1990 | | 2021 | | 1990–2021, AAPC (%),  (95% CI) |
| --- | --- | --- | --- | --- | --- | --- | --- | --- | --- | --- |
|  | Prevalence cases (95% UI) | ASPR per 100,000 (95% UI) | Prevalence cases (95% UI) | ASPR per 100,000 (95% UI) |  | YLDs cases (95% UI) | YLDs rate per 100,000 (95% UI) | YLDs cases (95% UI) | YLDs rate per 100,000 (95% UI) |  |
| Afghanistan | 680889 (577874 to 800728) | 30945.43 (26263.53 to 36391.93) | 1723617 (1512737 to 1969241) | 23954.79 (21023.98 to 27368.46) | -0.812 (-0.82 to -0.801) | 18366 (11073 to 27925) | 834.71 (503.26 to 1269.14) | 46864 (29551 to 70596) | 651.31 (410.70 to 981.15) | -0.786 (-0.795 to -0.776) |
| Albania | 312531 (243137 to 410760) | 37423.77 (29114.24 to 49186.11) | 172224 (144324 to 210173) | 28062.34 (23516.28 to 34245.87) | -0.94 (-0.961 to -0.912) | 5991 (3544 to 10085) | 717.34 (424.39 to 1207.67) | 2903 (1806 to 4586) | 473.01 (294.30 to 747.29) | -1.339 (-1.356 to -1.322) |
| Algeria | 2679898 (2099164 to 3581949) | 46115.47 (36122.25 to 61637.89) | 4055635 (3293173 to 5305335) | 36135.79 (29342.24 to 47270.65) | -0.785 (-0.79 to -0.779) | 57133 (33431 to 87999) | 983.14 (575.28 to 1514.29) | 89698 (53305 to 138678) | 799.21 (474.95 to 1235.62) | -0.663 (-0.676 to -0.655) |
| American Samoa | 4645 (3741 to 5937) | 38645.29 (31122.02 to 49388.65) | 4224 (3500 to 5193) | 35998.30 (29832.67 to 44263.23) | -0.232 (-0.238 to -0.226) | 107 (64 to 165) | 892.62 (530.87 to 1375.19) | 104 (63 to 157) | 889.07 (539.52 to 1340.91) | -0.013 (-0.018 to -0.009) |
| Andorra | 1325 (1044 to 1770) | 8879.31 (6998.59 to 11859.68) | 1554 (1253 to 1940) | 7775.67 (6271.08 to 9707.51) | -0.418 (-0.431 to -0.409) | 16 (8 to 28) | 107.36 (55.16 to 185.28) | 20 (11 to 33) | 99.67 (54.72 to 165.71) | -0.245 (-0.272 to -0.221) |
| Angola | 1362690 (1084846 to 1714692) | 59194.72 (47125.27 to 74485.58) | 4025443 (3091725 to 5087143) | 52448.63 (40282.95 to 66281.82) | -0.389 (-0.401 to -0.378) | 34451 (20164 to 51097) | 1496.55 (875.93 to 2219.65) | 87060 (53346 to 132187) | 1134.33 (695.06 to 1722.30) | -0.89 (-0.906 to -0.876) |
| Antigua and Barbuda | 6048 (5023 to 7419) | 37226.21 (30918.18 to 45664.68) | 8096 (6766 to 9972) | 33504.06 (27998.65 to 41269.41) | -0.344 (-0.351 to -0.335) | 159 (94 to 238) | 978.34 (576.28 to 1466.71) | 185 (108 to 297) | 765.58 (447.75 to 1227.20) | -0.787 (-0.797 to -0.778) |
| Argentina | 1254367 (669384 to 2163488) | 15627.27 (8339.38 to 26953.36) | 1988303 (823383 to 3726321) | 16727.68 (6927.16 to 31349.70) | 0.223 (0.201 to 0.241) | 12715 (4755 to 24453) | 158.40 (59.23 to 304.64) | 18031 (5020 to 40966) | 151.70 (42.23 to 344.65) | -0.137 (-0.149 to -0.126) |
| Armenia | 196899 (174295 to 219849) | 22734.00 (20124.20 to 25383.89) | 162162 (138097 to 198872) | 22008.82 (18742.68 to 26991.21) | -0.102 (-0.114 to -0.095) | 5581 (3288 to 8465) | 644.40 (379.61 to 977.32) | 3740 (2262 to 5573) | 507.63 (307.01 to 756.42) | -0.773 (-0.784 to -0.762) |
| Australia | 473544 (397599 to 575250) | 10609.24 (8907.76 to 12887.84) | 535948 (439571 to 657720) | 8896.40 (7296.60 to 10917.73) | -0.565 (-0.572 to -0.559) | 7110 (3761 to 11802) | 159.29 (84.26 to 264.42) | 8350 (4741 to 13276) | 138.60 (78.70 to 220.37) | -0.449 (-0.459 to -0.441) |
| Austria | 193668 (153904 to 266446) | 9786.75 (7777.33 to 13464.48) | 152728 (125966 to 188830) | 7734.99 (6379.62 to 9563.40) | -0.758 (-0.764 to -0.752) | 2399 (1231 to 4547) | 121.21 (62.19 to 229.76) | 1940 (1078 to 3185) | 98.27 (54.61 to 161.30) | -0.673 (-0.683 to -0.663) |
| Azerbaijan | 721295 (655391 to 790758) | 38465.40 (34950.84 to 42169.75) | 954548 (852429 to 1080608) | 34823.46 (31097.98 to 39422.33) | -0.32 (-0.329 to -0.312) | 24074 (14803 to 36524) | 1283.82 (789.42 to 1947.76) | 26077 (15608 to 39314) | 951.34 (569.41 to 1434.22) | -0.966 (-0.977 to -0.955) |
| Bahamas | 27797 (23099 to 34868) | 38323.40 (31845.64 to 48071.51) | 38661 (32031 to 48436) | 35857.42 (29707.86 to 44923.01) | -0.222 (-0.231 to -0.211) | 716 (442 to 1088) | 987.14 (609.03 to 1499.48) | 896 (546 to 1360) | 831.40 (506.36 to 1261.80) | -0.554 (-0.572 to -0.537) |
| Bahrain | 68082 (53796 to 85741) | 59048.65 (46657.99 to 74365.23) | 138686 (109529 to 176853) | 42538.83 (33595.49 to 54245.60) | -1.048 (-1.056 to -1.039) | 1226 (711 to 1976) | 1063.19 (616.40 to 1714.23) | 2437 (1427 to 3872) | 747.49 (437.57 to 1187.75) | -1.132 (-1.146 to -1.118) |
| Bangladesh | 16665581 (13631897 to 20023947) | 67692.40 (55370.16 to 81333.44) | 20628023 (16204711 to 26256837) | 44848.11 (35231.23 to 57085.92) | -1.336 (-1.352 to -1.319) | 371770 (225243 to 588196) | 1510.06 (914.89 to 2389.14) | 436333 (266024 to 675197) | 948.65 (578.37 to 1467.97) | -1.488 (-1.498 to -1.48) |
| Barbados | 23415 (20745 to 26383) | 34176.04 (30278.06 to 38506.85) | 21687 (18338 to 26548) | 30463.61 (25759.02 to 37292.70) | -0.371 (-0.373 to -0.368) | 677 (408 to 1031) | 987.46 (596.00 to 1504.32) | 582 (363 to 903) | 818.22 (510.04 to 1268.72) | -0.603 (-0.609 to -0.597) |
| Belarus | 588241 (505980 to 686316) | 23216.69 (19970.00 to 27087.50) | 423570 (358552 to 508984) | 19973.34 (16907.42 to 24001.01) | -0.486 (-0.495 to -0.474) | 16689 (10671 to 26643) | 658.68 (421.16 to 1051.54) | 10788 (6330 to 16739) | 508.70 (298.48 to 789.33) | -0.829 (-0.841 to -0.817) |
| Belgium | 208752 (177401 to 245638) | 8574.86 (7287.04 to 10090.03) | 176404 (143526 to 211179) | 7120.01 (5793.00 to 8523.58) | -0.596 (-0.603 to -0.59) | 3440 (2037 to 5549) | 141.30 (83.68 to 227.93) | 2898 (1695 to 4617) | 116.96 (68.42 to 186.36) | -0.604 (-0.613 to -0.597) |
| Belize | 18160 (15319 to 22374) | 43138.35 (36391.53 to 53149.30) | 49129 (40034 to 61475) | 40672.34 (33143.46 to 50893.46) | -0.186 (-0.194 to -0.178) | 565 (351 to 859) | 1341.46 (834.59 to 2040.83) | 1313 (809 to 1970) | 1086.73 (670.05 to 1630.88) | -0.682 (-0.696 to -0.667) |
| Benin | 564289 (512592 to 638102) | 51512.44 (46793.18 to 58250.62) | 2152466 (1828623 to 2519939) | 66349.73 (56367.29 to 77677.07) | 0.812 (0.793 to 0.827) | 22920 (14817 to 33837) | 2092.32 (1352.59 to 3088.93) | 57957 (35738 to 86833) | 1786.52 (1101.62 to 2676.63) | -0.509 (-0.524 to -0.494) |
| Bermuda | 5121 (4263 to 6369) | 29935.45 (24922.74 to 37231.22) | 3009 (2434 to 3741) | 22316.28 (18050.74 to 27750.47) | -0.943 (-0.949 to -0.937) | 114 (67 to 185) | 668.40 (391.80 to 1081.57) | 57 (34 to 86) | 419.42 (255.03 to 635.50) | -1.49 (-1.504 to -1.474) |
| Bhutan | 82128 (64786 to 107494) | 57367.10 (45253.33 to 75085.39) | 117006 (92918 to 143400) | 56516.19 (44881.11 to 69264.98) | -0.05 (-0.065 to -0.035) | 2612 (1442 to 4145) | 1824.17 (1007.21 to 2895.50) | 2757 (1638 to 4287) | 1331.56 (791.31 to 2070.91) | -1.036 (-1.058 to -1.011) |
| Bolivia (Plurinational State of) | 501665 (445370 to 570885) | 32791.43 (29111.68 to 37316.02) | 680446 (602219 to 757874) | 21823.33 (19314.42 to 24306.59) | -1.309 (-1.317 to -1.3) | 15116 (9535 to 22799) | 988.03 (623.28 to 1490.25) | 21076 (13298 to 31703) | 675.94 (426.51 to 1016.78) | -1.219 (-1.227 to -1.213) |
| Bosnia and Herzegovina | 301909 (257948 to 360708) | 25931.86 (22155.92 to 30982.26) | 170860 (142800 to 216743) | 23354.21 (19518.76 to 29625.74) | -0.333 (-0.348 to -0.318) | 8428 (4978 to 12953) | 723.89 (427.55 to 1112.59) | 3747 (2265 to 5770) | 512.17 (309.65 to 788.71) | -1.117 (-1.151 to -1.096) |
| Botswana | 124806 (112207 to 142130) | 38764.83 (34851.59 to 44145.81) | 238884 (195838 to 299390) | 35110.82 (28783.89 to 44003.90) | -0.322 (-0.338 to -0.309) | 2956 (1868 to 4462) | 918.19 (580.33 to 1386.01) | 5312 (3178 to 7963) | 780.82 (467.13 to 1170.41) | -0.517 (-0.536 to -0.5) |
| Brazil | 16019269 (13425274 to 19075758) | 41126.86 (34467.21 to 48973.90) | 20125547 (17140114 to 23833231) | 34276.17 (29191.63 to 40590.79) | -0.585 (-0.588 to -0.582) | 468118 (301272 to 691547) | 1201.82 (773.47 to 1775.44) | 514892 (316473 to 757316) | 876.92 (538.99 to 1289.80) | -1.01 (-1.016 to -1.005) |
| Brunei Darussalam | 10520 (8655 to 13090) | 15570.93 (12810.67 to 19374.10) | 17652 (14379 to 24519) | 14128.56 (11509.02 to 19625.09) | -0.319 (-0.327 to -0.312) | 133 (74 to 237) | 196.56 (109.06 to 350.62) | 253 (139 to 432) | 202.77 (111.09 to 345.86) | 0.098 (0.088 to 0.109) |
| Bulgaria | 522872 (450584 to 619635) | 25249.07 (21758.33 to 29921.68) | 359137 (302259 to 444069) | 25212.12 (21219.19 to 31174.47) | -0.003 (-0.013 to 0.005) | 14525 (8737 to 22237) | 701.40 (421.90 to 1073.81) | 8029 (4771 to 12688) | 563.65 (334.94 to 890.72) | -0.697 (-0.709 to -0.687) |
| Burkina Faso | 1023569 (919013 to 1159621) | 48900.02 (43904.96 to 55399.75) | 2577634 (2171220 to 3181239) | 46927.18 (39528.21 to 57916.13) | -0.12 (-0.141 to -0.103) | 34824 (21693 to 51426) | 1663.66 (1036.34 to 2456.81) | 80207 (49208 to 121016) | 1460.21 (895.86 to 2203.15) | -0.398 (-0.421 to -0.369) |
| Burundi | 474301 (376428 to 598476) | 37661.10 (29889.69 to 47521.06) | 1203802 (1020924 to 1455757) | 38490.74 (32643.33 to 46546.81) | 0.076 (0.054 to 0.096) | 11557 (7013 to 17441) | 917.67 (556.88 to 1384.85) | 27426 (16308 to 42633) | 876.91 (521.44 to 1363.17) | -0.15 (-0.176 to -0.125) |
| Cabo Verde | 38177 (30748 to 48692) | 48133.82 (38767.15 to 61391.98) | 63721 (50637 to 82964) | 42396.76 (33691.17 to 55200.46) | -0.414 (-0.423 to -0.406) | 975 (586 to 1504) | 1229.56 (738.95 to 1895.80) | 1445 (850 to 2201) | 961.36 (565.36 to 1464.24) | -0.791 (-0.799 to -0.783) |
| Cambodia | 1345351 (1164683 to 1613009) | 53720.61 (46506.45 to 64408.34) | 1874908 (1629544 to 2193740) | 41493.78 (36063.59 to 48549.88) | -0.83 (-0.838 to -0.822) | 46931 (30276 to 69161) | 1873.98 (1208.92 to 2761.64) | 49174 (31140 to 75188) | 1088.28 (689.16 to 1663.99) | -1.741 (-1.751 to -1.73) |
| Cameroon | 874790 (801861 to 951724) | 36765.30 (33700.28 to 39998.63) | 2944547 (2573302 to 3371235) | 37593.08 (32853.39 to 43040.60) | 0.072 (0.058 to 0.085) | 32858 (21033 to 47770) | 1380.92 (883.95 to 2007.65) | 88018 (56518 to 134352) | 1123.73 (721.56 to 1715.28) | -0.656 (-0.676 to -0.641) |
| Canada | 1150975 (594388 to 2014050) | 15646.46 (8080.17 to 27379.18) | 1029462 (640427 to 2051554) | 12412.24 (7721.63 to 24735.61) | -0.742 (-0.751 to -0.733) | 8179 (2017 to 19309) | 111.19 (27.42 to 262.49) | 8934 (3257 to 19872) | 107.72 (39.27 to 239.60) | -0.094 (-0.109 to -0.082) |
| Central African Republic | 254875 (213604 to 308005) | 39762.86 (33324.31 to 48051.79) | 530046 (448201 to 641630) | 38313.08 (32397.11 to 46378.66) | -0.123 (-0.133 to -0.114) | 7513 (4601 to 11379) | 1172.11 (717.86 to 1775.16) | 15586 (9661 to 23340) | 1126.59 (698.33 to 1687.04) | -0.136 (-0.146 to -0.125) |
| Chad | 768381 (639761 to 960400) | 57787.05 (48114.01 to 72228.12) | 2292097 (1855470 to 2772277) | 59258.23 (47969.99 to 71672.46) | 0.078 (0.072 to 0.083) | 24757 (14601 to 39026) | 1861.91 (1098.10 to 2934.99) | 66080 (41314 to 101150) | 1708.39 (1068.11 to 2615.05) | -0.278 (-0.288 to -0.27) |
| Chile | 428069 (322496 to 631366) | 11843.02 (8922.23 to 17467.46) | 377932 (300330 to 461207) | 8023.74 (6376.19 to 9791.73) | -1.243 (-1.254 to -1.231) | 5450 (2736 to 9533) | 150.79 (75.68 to 263.73) | 5493 (2885 to 9795) | 116.63 (61.26 to 207.95) | -0.819 (-0.83 to -0.808) |
| China | 84930455 (81456070 to 88712520) | 26358.68 (25280.38 to 27532.47) | 50337989 (48114980 to 52733632) | 15794.92 (15097.39 to 16546.61) | -1.643 (-1.65 to -1.635) | 2163408 (1419329 to 3176489) | 671.43 (440.50 to 985.84) | 1272261 (827113 to 1861791) | 399.21 (259.53 to 584.19) | -1.669 (-1.678 to -1.662) |
| Colombia | 1660315 (1329768 to 2129444) | 19117.45 (15311.42 to 24519.18) | 1585372 (1349126 to 1911173) | 12101.73 (10298.37 to 14588.68) | -1.473 (-1.488 to -1.457) | 27788 (15960 to 43335) | 319.96 (183.76 to 498.97) | 32610 (18390 to 50417) | 248.92 (140.38 to 384.85) | -0.812 (-0.825 to -0.8) |
| Comoros | 57846 (34331 to 82538) | 54905.59 (32585.38 to 78341.89) | 72956 (54417 to 95774) | 37370.66 (27874.53 to 49058.65) | -1.243 (-1.282 to -1.199) | 1090 (471 to 1894) | 1034.74 (447.27 to 1798.11) | 1555 (854 to 2495) | 796.49 (437.48 to 1277.93) | -0.84 (-0.882 to -0.793) |
| Congo | 308046 (260071 to 372663) | 54651.90 (46140.33 to 66115.78) | 702829 (602694 to 842958) | 49053.84 (42064.91 to 58834.10) | -0.35 (-0.362 to -0.339) | 10331 (6517 to 15027) | 1832.87 (1156.23 to 2665.92) | 21288 (13993 to 32324) | 1485.81 (976.67 to 2256.04) | -0.681 (-0.692 to -0.67) |
| Cook Islands | 1754 (1393 to 2316) | 38189.29 (30321.07 to 50420.73) | 1274 (1038 to 1593) | 29568.02 (24106.65 to 36972.82) | -0.825 (-0.831 to -0.818) | 38 (23 to 58) | 827.29 (497.31 to 1254.47) | 27 (16 to 43) | 615.88 (370.64 to 1005.50) | -0.948 (-0.956 to -0.941) |
| Costa Rica | 178688 (128523 to 243429) | 22978.50 (16527.46 to 31303.97) | 239104 (178631 to 345616) | 18470.34 (13798.86 to 26698.14) | -0.7 (-0.704 to -0.694) | 1997 (1032 to 3535) | 256.79 (132.77 to 454.63) | 2845 (1511 to 4829) | 219.74 (116.70 to 373.00) | -0.498 (-0.504 to -0.493) |
| Croatia | 258421 (221800 to 307384) | 21522.55 (18472.64 to 25600.47) | 171538 (141244 to 209899) | 19122.51 (15745.46 to 23398.92) | -0.827 (-0.848 to -0.801) | 6763 (4088 to 10666) | 563.23 (340.46 to 888.32) | 3601 (2247 to 5700) | 401.38 (250.49 to 635.46) | -0.549 (-0.557 to -0.541) |
| Cuba | 1055283 (885449 to 1309958) | 34439.97 (28897.29 to 42751.50) | 762396 (652474 to 921510) | 30632.06 (26215.56 to 37025.06) | -0.387 (-0.394 to -0.381) | 27815 (17476 to 41703) | 907.76 (570.34 to 1361.01) | 17864 (10903 to 27385) | 717.76 (438.06 to 1100.28) | -1.097 (-1.118 to -1.08) |
| Cyprus | 23339 (17693 to 36699) | 11815.59 (8957.31 to 18578.87) | 29213 (23488 to 36746) | 8180.25 (6577.13 to 10289.38) | -0.381 (-0.387 to -0.375) | 281 (144 to 537) | 142.38 (72.79 to 271.65) | 377 (201 to 668) | 105.70 (56.32 to 186.99) | -0.759 (-0.765 to -0.753) |
| Czechia | 576526 (490005 to 682424) | 22393.08 (19032.50 to 26506.29) | 439056 (364755 to 538275) | 19140.15 (15901.08 to 23465.49) | -1.163 (-1.178 to -1.148) | 15091 (8926 to 23146) | 586.16 (346.70 to 899.03) | 9124 (5433 to 14863) | 397.76 (236.83 to 647.94) | -0.957 (-0.968 to -0.947) |
| Côte d'Ivoire | 1722896 (1473185 to 2027950) | 62960.82 (53835.49 to 74108.57) | 3243632 (2779831 to 3790848) | 48711.83 (41746.61 to 56929.74) | -0.504 (-0.513 to -0.496) | 51436 (31066 to 77126) | 1879.65 (1135.26 to 2818.45) | 105790 (63870 to 156890) | 1588.71 (959.17 to 2356.12) | -1.239 (-1.254 to -1.227) |
| Democratic People's Republic of Korea | 1810344 (1500183 to 2219997) | 31923.50 (26454.14 to 39147.29) | 1777241 (1503447 to 2056299) | 26969.37 (22814.60 to 31204.05) | -0.547 (-0.559 to -0.538) | 39992 (24191 to 63091) | 705.22 (426.57 to 1112.54) | 39017 (23479 to 59526) | 592.08 (356.30 to 903.30) | -0.563 (-0.573 to -0.556) |
| Democratic Republic of the Congo | 6347384 (5530279 to 7225784) | 74372.16 (64798.15 to 84664.35) | 9848064 (8128220 to 12331431) | 46218.80 (38147.25 to 57873.70) | -1.525 (-1.548 to -1.506) | 198062 (123273 to 303131) | 2320.68 (1444.38 to 3551.78) | 250369 (150536 to 377249) | 1175.03 (706.49 to 1770.50) | -2.179 (-2.206 to -2.152) |
| Denmark | 133240 (104522 to 164419) | 10204.65 (8005.22 to 12592.65) | 101325 (80707 to 151462) | 8022.23 (6389.84 to 11991.72) | -0.774 (-0.781 to -0.768) | 1557 (815 to 2737) | 119.25 (62.40 to 209.59) | 1229 (651 to 2060) | 97.27 (51.55 to 163.11) | -0.655 (-0.664 to -0.647) |
| Djibouti | 52755 (40015 to 68474) | 53669.94 (40709.19 to 69662.02) | 120726 (93978 to 156300) | 37575.10 (29249.96 to 48647.03) | -1.131 (-1.154 to -1.106) | 929 (555 to 1479) | 945.11 (564.69 to 1504.96) | 2455 (1400 to 3766) | 764.05 (435.84 to 1172.22) | -0.657 (-0.685 to -0.625) |
| Dominica | 6498 (5630 to 7727) | 38141.79 (33051.69 to 45355.94) | 5606 (4978 to 6449) | 34106.03 (30282.59 to 39235.00) | -0.359 (-0.366 to -0.353) | 198 (122 to 292) | 1164.08 (714.35 to 1713.61) | 162 (99 to 238) | 982.79 (601.87 to 1446.52) | -0.544 (-0.548 to -0.54) |
| Dominican Republic | 760037 (658775 to 917852) | 40257.85 (34894.18 to 48617.04) | 979801 (857439 to 1147368) | 33823.40 (29599.38 to 39607.91) | -0.578 (-0.593 to -0.563) | 23852 (14801 to 35839) | 1263.38 (783.96 to 1898.31) | 26859 (16937 to 41151) | 927.20 (584.68 to 1420.57) | -0.999 (-1.013 to -0.986) |
| Ecuador | 454508 (378786 to 554350) | 18021.09 (15018.71 to 21979.76) | 569296 (499546 to 644991) | 12072.70 (10593.56 to 13677.93) | -1.288 (-1.295 to -1.279) | 8842 (5021 to 14376) | 350.57 (199.07 to 570.00) | 11338 (7174 to 17712) | 240.44 (152.13 to 375.62) | -1.215 (-1.229 to -1.201) |
| Egypt | 5019132 (4313304 to 5922055) | 38274.95 (32892.44 to 45160.47) | 6774108 (5932381 to 7824775) | 26106.75 (22862.82 to 30155.93) | -1.239 (-1.262 to -1.216) | 112577 (67774 to 173705) | 858.49 (516.83 to 1324.64) | 133583 (84343 to 202527) | 514.82 (325.05 to 780.52) | -1.631 (-1.651 to -1.617) |
| El Salvador | 166809 (143081 to 191700) | 12726.08 (10915.78 to 14625.02) | 166515 (141457 to 194444) | 9374.33 (7963.64 to 10946.68) | -0.99 (-1.003 to -0.978) | 3616 (2170 to 5767) | 275.85 (165.57 to 439.94) | 3233 (1937 to 4908) | 182.01 (109.05 to 276.33) | -1.34 (-1.352 to -1.33) |
| Equatorial Guinea | 60552 (50256 to 72413) | 61308.56 (50883.38 to 73317.08) | 172602 (138901 to 222643) | 47356.98 (38110.35 to 61086.62) | -0.825 (-0.832 to -0.819) | 1920 (1148 to 2912) | 1943.99 (1162.58 to 2948.57) | 4415 (2648 to 6694) | 1211.28 (726.41 to 1836.69) | -1.51 (-1.528 to -1.492) |
| Eritrea | 459607 (360605 to 569194) | 58281.91 (45727.69 to 72178.36) | 685552 (546537 to 867760) | 41417.45 (33018.88 to 52425.53) | -1.097 (-1.109 to -1.084) | 8930 (5200 to 14723) | 1132.45 (659.39 to 1867.01) | 14428 (8741 to 22526) | 871.63 (528.08 to 1360.89) | -0.839 (-0.846 to -0.831) |
| Estonia | 88183 (76793 to 104391) | 23142.15 (20153.12 to 27395.79) | 51776 (44309 to 61891) | 18599.88 (15917.54 to 22233.81) | -0.702 (-0.711 to -0.693) | 2480 (1510 to 3739) | 650.91 (396.30 to 981.30) | 1263 (783 to 1964) | 453.76 (281.12 to 705.46) | -1.152 (-1.166 to -1.14) |
| Eswatini | 62007 (53595 to 72698) | 31815.36 (27499.28 to 37301.22) | 91015 (80713 to 104194) | 28877.82 (25609.28 to 33059.33) | -0.309 (-0.32 to -0.296) | 1703 (1024 to 2559) | 873.61 (525.22 to 1312.83) | 2583 (1577 to 3865) | 819.51 (500.34 to 1226.47) | -0.204 (-0.218 to -0.19) |
| Ethiopia | 3437139 (3239073 to 3696827) | 30461.48 (28706.13 to 32762.95) | 6430406 (6100092 to 6785590) | 23198.45 (22006.81 to 24479.82) | -0.87 (-0.895 to -0.842) | 110361 (71847 to 161869) | 978.07 (636.74 to 1434.55) | 186876 (124313 to 275446) | 674.18 (448.47 to 993.71) | -1.188 (-1.207 to -1.165) |
| Fiji | 74492 (67271 to 82107) | 38104.65 (34411.11 to 42000.19) | 86186 (74934 to 101811) | 37737.41 (32810.65 to 44578.75) | -0.036 (-0.045 to -0.029) | 2019 (1229 to 3045) | 1032.74 (628.83 to 1557.54) | 2239 (1329 to 3471) | 980.23 (582.09 to 1519.73) | -0.169 (-0.176 to -0.163) |
| Finland | 119251 (97935 to 144635) | 9439.50 (7752.17 to 11448.75) | 81055 (65249 to 98081) | 7131.85 (5741.11 to 8629.90) | -0.903 (-0.908 to -0.898) | 1681 (947 to 2798) | 133.10 (74.95 to 221.51) | 1169 (634 to 1897) | 102.81 (55.75 to 166.88) | -0.826 (-0.835 to -0.819) |
| France | 1181087 (1004084 to 1387661) | 8161.23 (6938.15 to 9588.64) | 946372 (788558 to 1134207) | 6665.84 (5554.27 to 7988.88) | -0.649 (-0.655 to -0.642) | 23240 (13388 to 37243) | 160.59 (92.51 to 257.35) | 17530 (10622 to 27052) | 123.47 (74.82 to 190.54) | -0.844 (-0.852 to -0.837) |
| Gabon | 136824 (117275 to 157615) | 61805.37 (52974.97 to 71197.17) | 283067 (244740 to 338700) | 57820.07 (49991.26 to 69183.86) | -0.202 (-0.216 to -0.189) | 5405 (3608 to 7959) | 2441.32 (1629.72 to 3595.22) | 9666 (6188 to 14178) | 1974.39 (1264.07 to 2896.04) | -0.666 (-0.684 to -0.649) |
| Gambia | 131841 (116333 to 151658) | 58097.46 (51263.72 to 66829.93) | 352339 (309641 to 411313) | 57292.92 (50349.87 to 66882.52) | -0.041 (-0.053 to -0.029) | 4913 (3090 to 7341) | 2165.09 (1361.76 to 3234.82) | 12032 (7820 to 17262) | 1956.55 (1271.66 to 2806.94) | -0.324 (-0.336 to -0.313) |
| Georgia | 387288 (336993 to 457833) | 28215.40 (24551.21 to 33354.81) | 195608 (173236 to 219072) | 24855.93 (22013.01 to 27837.49) | -0.401 (-0.408 to -0.395) | 10019 (5934 to 15081) | 729.95 (432.32 to 1098.73) | 5499 (3567 to 8221) | 698.80 (453.29 to 1044.61) | -0.139 (-0.151 to -0.127) |
| Germany | 3234041 (1583478 to 5924856) | 16640.10 (8147.47 to 30485.15) | 1952444 (1139272 to 4038892) | 11464.25 (6689.51 to 23715.33) | -1.174 (-1.208 to -1.136) | 22277 (6621 to 52073) | 114.62 (34.07 to 267.93) | 14858 (5219 to 33755) | 87.24 (30.64 to 198.20) | -0.872 (-0.904 to -0.838) |
| Ghana | 2450678 (2061758 to 2870376) | 69486.39 (58458.97 to 81386.48) | 5204981 (4232931 to 6296688) | 57007.66 (46361.26 to 68964.59) | -0.635 (-0.655 to -0.617) | 71160 (42120 to 107100) | 2017.67 (1194.27 to 3036.71) | 138754 (84809 to 207295) | 1519.70 (928.87 to 2270.41) | -0.902 (-0.923 to -0.877) |
| Greece | 273839 (217351 to 394215) | 10867.84 (8625.97 to 15645.19) | 217041 (174897 to 280057) | 10013.24 (8068.88 to 12920.47) | -0.267 (-0.274 to -0.259) | 3568 (1846 to 5959) | 141.62 (73.27 to 236.48) | 2908 (1588 to 4921) | 134.14 (73.25 to 227.04) | -0.175 (-0.189 to -0.164) |
| Greenland | 2396 (1761 to 3601) | 16262.88 (11953.89 to 24447.10) | 1420 (1170 to 1715) | 11159.22 (9190.40 to 13474.96) | -1.214 (-1.235 to -1.184) | 33 (17 to 59) | 224.98 (115.21 to 398.52) | 22 (13 to 38) | 175.04 (100.88 to 298.51) | -0.8 (-0.824 to -0.777) |
| Grenada | 7009 (6494 to 7535) | 36011.73 (33364.65 to 38709.96) | 8412 (7213 to 10023) | 32840.51 (28160.02 to 39128.03) | -0.298 (-0.3 to -0.295) | 250 (151 to 369) | 1286.77 (775.04 to 1893.75) | 247 (155 to 365) | 965.40 (606.68 to 1426.78) | -0.92 (-0.927 to -0.913) |
| Guam | 12250 (9561 to 15973) | 35107.49 (27401.06 to 45778.67) | 11713 (9439 to 14759) | 32466.18 (26162.73 to 40907.84) | -0.26 (-0.276 to -0.242) | 238 (142 to 373) | 680.78 (407.61 to 1069.96) | 234 (147 to 360) | 649.58 (408.30 to 997.44) | -0.158 (-0.176 to -0.137) |
| Guatemala | 750342 (653071 to 879111) | 40842.21 (35547.61 to 47851.32) | 1480885 (1304019 to 1709567) | 33691.90 (29667.97 to 38894.67) | -0.615 (-0.622 to -0.609) | 23493 (15152 to 34591) | 1278.73 (824.77 to 1882.83) | 44864 (28836 to 66467) | 1020.70 (656.05 to 1512.20) | -0.716 (-0.732 to -0.702) |
| Guinea | 689772 (594664 to 810555) | 50568.37 (43595.84 to 59423.16) | 1575032 (1391683 to 1819852) | 47460.77 (41935.87 to 54837.99) | -0.212 (-0.221 to -0.202) | 23365 (14905 to 34828) | 1712.91 (1092.68 to 2553.27) | 47947 (31137 to 70113) | 1444.79 (938.26 to 2112.72) | -0.555 (-0.569 to -0.542) |
| Guinea-Bissau | 140794 (115764 to 172989) | 60357.95 (49627.69 to 74159.81) | 297797 (241479 to 372037) | 56689.57 (45968.70 to 70822.20) | -0.203 (-0.209 to -0.197) | 4649 (2972 to 6937) | 1992.92 (1274.14 to 2973.83) | 8490 (5136 to 13483) | 1616.11 (977.71 to 2566.60) | -0.673 (-0.684 to -0.661) |
| Guyana | 107003 (90598 to 131629) | 52387.86 (44356.01 to 64444.77) | 78620 (67809 to 94442) | 38691.86 (33371.56 to 46478.73) | -0.973 (-0.996 to -0.936) | 2584 (1582 to 3892) | 1265.35 (774.49 to 1905.28) | 2079 (1309 to 3214) | 1023.32 (644.31 to 1581.54) | -0.701 (-0.732 to -0.669) |
| Haiti | 864781 (772639 to 975668) | 56205.86 (50217.14 to 63412.89) | 2101957 (1804393 to 2467979) | 59377.48 (50971.70 to 69717.11) | 0.179 (0.158 to 0.197) | 38565 (25014 to 55066) | 2506.51 (1625.77 to 3578.96) | 58394 (36856 to 85574) | 1649.55 (1041.14 to 2417.35) | -1.353 (-1.369 to -1.339) |
| Honduras | 242082 (213022 to 273921) | 22945.60 (20191.14 to 25963.40) | 467581 (410423 to 530967) | 16472.89 (14459.23 to 18705.98) | -1.079 (-1.087 to -1.069) | 5006 (3077 to 8078) | 474.53 (291.65 to 765.63) | 10139 (6347 to 15509) | 357.18 (223.61 to 546.40) | -0.916 (-0.923 to -0.91) |
| Hungary | 600771 (515068 to 708039) | 23701.59 (20320.42 to 27933.50) | 435200 (355438 to 543808) | 20347.24 (16618.08 to 25425.09) | -0.49 (-0.501 to -0.48) | 16243 (9728 to 24598) | 640.80 (383.80 to 970.43) | 9406 (5300 to 14801) | 439.77 (247.79 to 692.02) | -1.206 (-1.215 to -1.198) |
| Iceland | 5264 (4320 to 6443) | 8115.79 (6660.06 to 9933.09) | 5239 (4344 to 6372) | 6578.64 (5454.72 to 8001.69) | -0.67 (-0.677 to -0.663) | 72 (42 to 125) | 111.45 (64.28 to 192.28) | 75 (43 to 122) | 94.46 (53.56 to 153.30) | -0.527 (-0.535 to -0.52) |
| India | 123299327 (120778050 to 125734945) | 61067.26 (59818.53 to 62273.56) | 209965847 (202245584 to 217608487) | 55504.05 (53463.21 to 57524.37) | -0.308 (-0.311 to -0.306) | 5272201 (3575803 to 7394444) | 2611.20 (1771.01 to 3662.29) | 6542738 (4353799 to 9307020) | 1729.56 (1150.92 to 2460.29) | -1.321 (-1.326 to -1.316) |
| Indonesia | 17708841 (15271678 to 20607758) | 36951.51 (31866.09 to 43000.44) | 21221349 (18189962 to 24752822) | 28184.81 (24158.72 to 32875.08) | -0.867 (-0.894 to -0.842) | 322002 (191256 to 495818) | 671.89 (399.08 to 1034.58) | 368934 (223202 to 571604) | 489.99 (296.44 to 759.17) | -1.011 (-1.02 to -1.003) |
| Iran (Islamic Republic of) | 4543847 (4114423 to 5058424) | 35926.96 (32531.62 to 39995.58) | 6522118 (5555907 to 7602339) | 28057.62 (23901.06 to 32704.65) | -0.785 (-0.797 to -0.775) | 119154 (73151 to 181724) | 942.12 (578.38 to 1436.84) | 176385 (111939 to 268375) | 758.80 (481.55 to 1154.53) | -0.692 (-0.703 to -0.681) |
| Iraq | 1638303 (1337459 to 2039790) | 40375.21 (32961.04 to 50269.68) | 3253864 (2706477 to 4075773) | 30864.21 (25672.03 to 38660.35) | -0.857 (-0.865 to -0.85) | 38361 (22893 to 59410) | 945.38 (564.20 to 1464.13) | 75436 (45091 to 116913) | 715.54 (427.71 to 1108.97) | -0.885 (-0.899 to -0.868) |
| Ireland | 92291 (71109 to 136150) | 10451.35 (8052.62 to 15418.16) | 86939 (70077 to 109443) | 7451.65 (6006.37 to 9380.48) | -1.089 (-1.098 to -1.079) | 1092 (596 to 1899) | 123.67 (67.52 to 215.06) | 1081 (567 to 1776) | 92.62 (48.64 to 152.23) | -0.937 (-0.949 to -0.925) |
| Israel | 153026 (117552 to 233826) | 12544.26 (9636.26 to 19167.81) | 213201 (170867 to 279250) | 9584.47 (7681.35 to 12553.70) | -0.859 (-0.865 to -0.853) | 1882 (936 to 3323) | 154.30 (76.69 to 272.43) | 2696 (1468 to 4721) | 121.18 (65.99 to 212.25) | -0.775 (-0.783 to -0.768) |
| Italy | 1463020 (1081109 to 2172832) | 10236.03 (7563.99 to 15202.24) | 985984 (761332 to 1367755) | 8126.24 (6274.72 to 11272.70) | -0.735 (-0.744 to -0.726) | 17598 (9368 to 29466) | 123.13 (65.54 to 206.16) | 12698 (6758 to 21599) | 104.66 (55.70 to 178.01) | -0.518 (-0.53 to -0.508) |
| Jamaica | 227608 (191035 to 284410) | 38200.24 (32062.01 to 47733.50) | 276388 (228441 to 351518) | 35795.93 (29586.14 to 45526.25) | -0.209 (-0.216 to -0.202) | 6341 (3768 to 9646) | 1064.17 (632.42 to 1618.84) | 6778 (4151 to 10213) | 877.88 (537.57 to 1322.69) | -0.62 (-0.626 to -0.614) |
| Japan | 6366292 (4376008 to 8999957) | 19830.87 (13631.18 to 28034.68) | 3920744 (2680682 to 5767050) | 15767.27 (10780.36 to 23192.20) | -0.746 (-0.766 to -0.726) | 60924 (30879 to 109161) | 189.78 (96.19 to 340.03) | 38806 (20281 to 69830) | 156.06 (81.56 to 280.82) | -0.64 (-0.656 to -0.624) |
| Jordan | 358227 (302144 to 432423) | 42955.73 (36230.66 to 51852.68) | 1274895 (1152567 to 1437946) | 41138.76 (37191.45 to 46400.16) | -0.165 (-0.199 to -0.133) | 9065 (5203 to 13723) | 1087.01 (623.91 to 1645.57) | 32583 (20206 to 49724) | 1051.39 (652.01 to 1604.52) | -0.107 (-0.117 to -0.097) |
| Kazakhstan | 1850536 (1672433 to 2062968) | 44971.42 (40643.20 to 50133.90) | 1802250 (1522071 to 2256781) | 38056.07 (32139.84 to 47653.89) | -0.535 (-0.544 to -0.53) | 56065 (34020 to 84374) | 1362.48 (826.75 to 2050.44) | 46708 (28862 to 70043) | 986.29 (609.44 to 1479.01) | -1.039 (-1.053 to -1.026) |
| Kenya | 1322960 (1240417 to 1395185) | 25542.49 (23948.84 to 26936.95) | 3019701 (2816778 to 3231898) | 22751.32 (21222.44 to 24350.08) | -0.378 (-0.397 to -0.362) | 33006 (21877 to 46991) | 637.26 (422.39 to 907.25) | 82164 (55899 to 117960) | 619.05 (421.16 to 888.74) | -0.094 (-0.119 to -0.075) |
| Kiribati | 9637 (7876 to 12109) | 51552.31 (42134.53 to 64776.82) | 15035 (12365 to 18858) | 47027.41 (38673.91 to 58983.76) | -0.298 (-0.304 to -0.292) | 265 (164 to 388) | 1418.84 (875.46 to 2078.01) | 408 (261 to 632) | 1275.99 (816.34 to 1977.31) | -0.346 (-0.353 to -0.34) |
| Kuwait | 171343 (145389 to 204100) | 41343.31 (35081.05 to 49247.21) | 395026 (329234 to 499675) | 27024.16 (22523.23 to 34183.30) | -1.37 (-1.399 to -1.34) | 2853 (1642 to 4320) | 688.34 (396.26 to 1042.45) | 8477 (5070 to 13340) | 579.90 (346.84 to 912.58) | -0.54 (-0.567 to -0.515) |
| Kyrgyzstan | 422678 (381073 to 473718) | 40324.52 (36355.28 to 45193.86) | 619810 (554912 to 707770) | 35949.45 (32185.34 to 41051.23) | -0.369 (-0.377 to -0.362) | 13077 (8395 to 19427) | 1247.56 (800.95 to 1853.40) | 17892 (11494 to 26457) | 1037.75 (666.67 to 1534.55) | -0.59 (-0.601 to -0.58) |
| Lao People's Democratic Republic | 454620 (390746 to 544708) | 46978.20 (40377.73 to 56287.50) | 945288 (822663 to 1093266) | 47637.52 (41457.88 to 55094.83) | 0.049 (0.038 to 0.058) | 14081 (8856 to 21810) | 1455.06 (915.13 to 2253.73) | 19459 (12138 to 28530) | 980.62 (611.70 to 1437.78) | -1.27 (-1.277 to -1.262) |
| Latvia | 152008 (130445 to 176597) | 23454.48 (20127.34 to 27248.53) | 79906 (67678 to 94658) | 20505.42 (17367.35 to 24291.15) | -0.43 (-0.445 to -0.419) | 4247 (2617 to 6506) | 655.33 (403.73 to 1003.91) | 1997 (1211 to 3096) | 512.41 (310.68 to 794.48) | -0.787 (-0.801 to -0.774) |
| Lebanon | 242385 (221561 to 266506) | 32345.11 (29566.28 to 35563.97) | 355057 (310392 to 403371) | 23932.13 (20921.57 to 27188.68) | -0.962 (-0.97 to -0.955) | 7405 (4597 to 10882) | 988.19 (613.49 to 1452.11) | 11476 (7215 to 17061) | 773.52 (486.34 to 1149.96) | -0.786 (-0.794 to -0.778) |
| Lesotho | 123736 (109760 to 141112) | 32705.35 (29011.09 to 37298.05) | 146966 (132768 to 160766) | 28993.77 (26192.65 to 31716.13) | -0.388 (-0.397 to -0.376) | 3789 (2307 to 5875) | 1001.44 (609.69 to 1552.84) | 4594 (2906 to 6983) | 906.30 (573.32 to 1377.58) | -0.32 (-0.328 to -0.312) |
| Liberia | 386021 (328426 to 462760) | 69575.48 (59194.81 to 83406.69) | 1008327 (850949 to 1216564) | 72264.28 (60985.43 to 87188.11) | 0.136 (0.12 to 0.151) | 18618 (12166 to 26990) | 3355.60 (2192.83 to 4864.54) | 47342 (30402 to 68163) | 3392.90 (2178.87 to 4885.07) | 0.034 (0.025 to 0.04) |
| Libya | 378180 (294611 to 500039) | 41524.73 (32348.79 to 54905.10) | 652788 (543641 to 811555) | 32817.25 (27330.16 to 40798.85) | -0.749 (-0.771 to -0.726) | 7815 (4647 to 12493) | 858.06 (510.28 to 1371.74) | 15591 (9092 to 24468) | 783.78 (457.08 to 1230.08) | -0.27 (-0.309 to -0.226) |
| Lithuania | 210363 (182409 to 245778) | 22818.61 (19786.32 to 26660.13) | 116343 (98241 to 139385) | 20306.81 (17147.13 to 24328.51) | -0.376 (-0.383 to -0.37) | 5864 (3673 to 8787) | 636.13 (398.37 to 953.15) | 2883 (1759 to 4559) | 503.14 (307.05 to 795.82) | -0.755 (-0.766 to -0.746) |
| Luxembourg | 9819 (7770 to 15046) | 10104.03 (7995.64 to 15483.08) | 11575 (9352 to 14310) | 7435.10 (6007.13 to 9191.84) | -0.986 (-0.992 to -0.981) | 114 (59 to 190) | 117.71 (61.08 to 195.46) | 142 (78 to 240) | 91.04 (50.05 to 154.42) | -0.82 (-0.834 to -0.807) |
| Madagascar | 1145269 (1001752 to 1316454) | 42523.70 (37194.92 to 48879.77) | 2629150 (2291194 to 3162635) | 36297.17 (31631.47 to 43662.30) | -0.506 (-0.516 to -0.494) | 36851 (22877 to 54608) | 1368.27 (849.43 to 2027.60) | 76178 (45287 to 119172) | 1051.68 (625.21 to 1645.25) | -0.851 (-0.863 to -0.839) |
| Malawi | 1698544 (1584480 to 1864335) | 75226.56 (70174.80 to 82569.24) | 2990278 (2267426 to 3916799) | 59694.85 (45264.57 to 78190.97) | -0.761 (-0.793 to -0.741) | 71955 (45590 to 103880) | 3186.80 (2019.15 to 4600.72) | 53114 (31918 to 84724) | 1060.31 (637.18 to 1691.34) | -3.487 (-3.537 to -3.437) |
| Malaysia | 1767078 (1626812 to 1901065) | 39439.76 (36309.13 to 42430.23) | 5446358 (4205356 to 6881632) | 64632.10 (49905.09 to 81664.54) | 1.622 (1.576 to 1.656) | 43327 (26457 to 69405) | 967.03 (590.50 to 1549.07) | 76012 (41927 to 123589) | 902.04 (497.55 to 1466.64) | -0.219 (-0.247 to -0.193) |
| Maldives | 27781 (24367 to 32079) | 58365.67 (51193.29 to 67396.72) | 69934 (59578 to 83154) | 60710.58 (51720.63 to 72187.55) | 0.117 (0.094 to 0.137) | 1117 (740 to 1597) | 2347.29 (1555.58 to 3354.61) | 2190 (1402 to 3213) | 1901.31 (1217.36 to 2788.91) | -0.69 (-0.703 to -0.681) |
| Mali | 1163283 (1014085 to 1346760) | 60621.72 (52846.66 to 70183.22) | 3272632 (2830495 to 3952577) | 60110.75 (51989.70 to 72599.79) | -0.022 (-0.032 to -0.012) | 45259 (28616 to 68202) | 2358.59 (1491.27 to 3554.20) | 113832 (72633 to 170426) | 2090.83 (1334.11 to 3130.34) | -0.382 (-0.397 to -0.367) |
| Malta | 11380 (8849 to 16683) | 12000.10 (9330.57 to 17591.19) | 8483 (6916 to 10504) | 8998.55 (7335.67 to 11141.99) | -0.92 (-0.927 to -0.915) | 145 (79 to 251) | 152.76 (83.55 to 264.97) | 110 (63 to 192) | 116.58 (66.49 to 203.67) | -0.872 (-0.88 to -0.864) |
| Marshall Islands | 3607 (3185 to 4191) | 36888.41 (32575.93 to 42861.53) | 5304 (4583 to 6366) | 35759.98 (30900.89 to 42923.75) | -0.1 (-0.102 to -0.098) | 117 (71 to 180) | 1199.42 (730.84 to 1842.62) | 165 (98 to 255) | 1112.75 (663.73 to 1717.09) | -0.241 (-0.251 to -0.231) |
| Mauritania | 269083 (218580 to 326441) | 57243.80 (46500.05 to 69446.03) | 542062 (434191 to 673146) | 50497.85 (40448.65 to 62709.49) | -0.398 (-0.408 to -0.389) | 7885 (4982 to 12019) | 1677.33 (1059.93 to 2556.92) | 13228 (8129 to 20636) | 1232.27 (757.33 to 1922.40) | -0.98 (-0.996 to -0.966) |
| Mauritius | 110726 (92462 to 136231) | 37061.02 (30947.88 to 45597.79) | 110775 (90244 to 140541) | 35000.10 (28513.33 to 44404.91) | -0.183 (-0.188 to -0.179) | 2793 (1612 to 4364) | 934.72 (539.51 to 1460.66) | 2451 (1518 to 3640) | 774.36 (479.64 to 1149.95) | -0.602 (-0.609 to -0.594) |
| Mexico | 2577116 (2499608 to 2653873) | 11771.33 (11417.30 to 12121.93) | 3801861 (3655196 to 3931315) | 10848.83 (10430.31 to 11218.23) | -0.268 (-0.278 to -0.261) | 61132 (39892 to 90041) | 279.23 (182.21 to 411.28) | 86900 (56077 to 127207) | 247.98 (160.02 to 362.99) | -0.379 (-0.39 to -0.368) |
| Micronesia (Federated States of) | 10824 (8925 to 13373) | 46679.21 (38491.03 to 57670.70) | 10665 (8805 to 13203) | 40782.18 (33671.08 to 50490.01) | -0.433 (-0.437 to -0.428) | 289 (181 to 461) | 1245.09 (780.13 to 1990.15) | 280 (177 to 424) | 1069.47 (674.98 to 1622.40) | -0.488 (-0.494 to -0.482) |
| Monaco | 513 (401 to 713) | 7366.16 (5747.84 to 10230.28) | 450 (360 to 561) | 6296.68 (5041.68 to 7854.56) | -0.506 (-0.515 to -0.497) | 6 (3 to 9) | 82.04 (43.59 to 135.47) | 5 (3 to 8) | 71.85 (37.14 to 114.57) | -0.426 (-0.438 to -0.414) |
| Mongolia | 181276 (167235 to 195459) | 35422.14 (32678.47 to 38193.47) | 233270 (209225 to 256080) | 27644.29 (24794.85 to 30347.47) | -0.802 (-0.824 to -0.789) | 8197 (5286 to 11874) | 1601.77 (1032.93 to 2320.29) | 9736 (5830 to 14583) | 1153.79 (690.92 to 1728.25) | -1.056 (-1.073 to -1.042) |
| Montenegro | 33250 (28411 to 39726) | 21303.26 (18203.05 to 25452.73) | 30992 (26172 to 38304) | 21518.97 (18171.92 to 26595.58) | 0.029 (0.025 to 0.034) | 863 (529 to 1301) | 552.61 (338.71 to 833.47) | 667 (405 to 1033) | 463.32 (281.51 to 717.30) | -0.571 (-0.584 to -0.558) |
| Morocco | 2869671 (2266720 to 3731437) | 45567.40 (35993.16 to 59251.35) | 3368181 (2748288 to 4186713) | 34774.50 (28374.46 to 43225.36) | -0.863 (-0.869 to -0.858) | 65368 (39835 to 100252) | 1037.98 (632.54 to 1591.89) | 78982 (48538 to 126014) | 815.45 (501.12 to 1301.02) | -0.772 (-0.78 to -0.764) |
| Mozambique | 1681708 (1531203 to 1872049) | 53293.60 (48524.09 to 59325.55) | 4338116 (4021406 to 4718742) | 57138.47 (52967.00 to 62151.79) | 0.226 (0.22 to 0.233) | 87667 (59693 to 127016) | 2778.19 (1891.67 to 4025.16) | 280915 (198888 to 389145) | 3700.01 (2619.61 to 5125.53) | 0.945 (0.924 to 0.962) |
| Myanmar | 5110341 (4459413 to 5995639) | 49033.56 (42787.92 to 57527.96) | 8662667 (7357663 to 10370023) | 57300.68 (48668.51 to 68594.27) | 0.511 (0.49 to 0.526) | 166132 (106321 to 241450) | 1594.03 (1020.14 to 2316.70) | 187792 (120566 to 284459) | 1242.18 (797.51 to 1881.60) | -0.803 (-0.81 to -0.797) |
| Namibia | 258581 (198212 to 314327) | 76254.22 (58451.58 to 92693.31) | 202010 (165999 to 261536) | 30546.74 (25101.41 to 39548.00) | -2.909 (-2.94 to -2.883) | 3750 (1753 to 6660) | 1105.88 (516.88 to 1963.89) | 3573 (2096 to 5585) | 540.25 (316.92 to 844.55) | -2.273 (-2.303 to -2.245) |
| Nauru | 1166 (932 to 1516) | 47894.22 (38287.04 to 62289.11) | 1224 (995 to 1560) | 42971.45 (34954.75 to 54780.71) | -0.351 (-0.355 to -0.346) | 27 (16 to 42) | 1122.67 (672.12 to 1709.26) | 29 (18 to 46) | 1014.64 (630.32 to 1603.69) | -0.322 (-0.329 to -0.315) |
| Nepal | 3628936 (3249168 to 4083738) | 79450.86 (71136.34 to 89408.17) | 4583004 (3742442 to 5668652) | 50522.02 (41255.85 to 62489.96) | -1.465 (-1.499 to -1.435) | 101234 (58543 to 154207) | 2216.39 (1281.73 to 3376.16) | 92842 (57882 to 139896) | 1023.47 (638.08 to 1542.18) | -2.451 (-2.474 to -2.421) |
| Netherlands | 381187 (307865 to 477396) | 9621.54 (7770.82 to 12049.96) | 289037 (232950 to 357447) | 7876.50 (6348.07 to 9740.73) | -0.65 (-0.664 to -0.64) | 4797 (2642 to 7608) | 121.07 (66.69 to 192.04) | 3712 (1988 to 6011) | 101.15 (54.18 to 163.82) | -0.579 (-0.594 to -0.567) |
| New Zealand | 93019 (79566 to 107664) | 10290.37 (8802.17 to 11910.49) | 101744 (84901 to 119882) | 8515.94 (7106.21 to 10034.10) | -0.607 (-0.613 to -0.601) | 1623 (922 to 2504) | 179.59 (102.04 to 276.98) | 1813 (1102 to 2840) | 151.79 (92.24 to 237.69) | -0.542 (-0.551 to -0.536) |
| Nicaragua | 190276 (158766 to 228089) | 21089.04 (17596.69 to 25280.00) | 290207 (250512 to 340999) | 16007.98 (13818.38 to 18809.71) | -0.887 (-0.893 to -0.881) | 3943 (2287 to 6339) | 437.06 (253.51 to 702.58) | 6960 (4216 to 10625) | 383.91 (232.57 to 586.11) | -0.416 (-0.422 to -0.411) |
| Niger | 1263377 (1078588 to 1481140) | 72626.75 (62003.92 to 85145.10) | 2756305 (2267339 to 3487823) | 51702.43 (42530.48 to 65424.18) | -1.099 (-1.119 to -1.078) | 39744 (22556 to 62663) | 2284.76 (1296.65 to 3602.27) | 74178 (46913 to 113633) | 1391.43 (880.00 to 2131.51) | -1.585 (-1.606 to -1.56) |
| Nigeria | 12772425 (11305862 to 14198865) | 63067.75 (55826.15 to 70111.24) | 34039806 (32428130 to 35723442) | 59281.35 (56474.57 to 62213.45) | -0.208 (-0.216 to -0.198) | 473442 (309198 to 688925) | 2337.76 (1526.76 to 3401.78) | 1045814 (685202 to 1542789) | 1821.32 (1193.30 to 2686.81) | -0.803 (-0.818 to -0.791) |
| Niue | 210 (167 to 267) | 42365.02 (33739.10 to 53841.56) | 141 (114 to 179) | 36659.05 (29633.93 to 46638.32) | -0.47 (-0.476 to -0.464) | 5 (3 to 8) | 996.16 (610.72 to 1559.80) | 3 (2 to 5) | 847.61 (515.76 to 1310.14) | -0.52 (-0.526 to -0.514) |
| North Macedonia | 116093 (103113 to 128492) | 22811.60 (20261.16 to 25247.98) | 107452 (93513 to 121985) | 20246.53 (17620.11 to 22984.76) | -0.388 (-0.393 to -0.384) | 4032 (2602 to 5889) | 792.31 (511.37 to 1157.17) | 3024 (1857 to 4779) | 569.81 (349.86 to 900.49) | -1.061 (-1.072 to -1.051) |
| Northern Mariana Islands | 5036 (3978 to 6553) | 36469.29 (28811.01 to 47457.36) | 3795 (3040 to 4756) | 33156.80 (26563.69 to 41551.47) | -0.307 (-0.317 to -0.297) | 97 (57 to 149) | 705.71 (412.76 to 1081.00) | 81 (49 to 130) | 709.54 (426.46 to 1137.10) | 0.033 (0.007 to 0.056) |
| Norway | 85737 (68855 to 108421) | 8123.08 (6523.65 to 10272.30) | 75026 (59976 to 96326) | 6194.76 (4952.15 to 7953.54) | -0.873 (-0.88 to -0.866) | 1141 (621 to 1915) | 108.08 (58.82 to 181.48) | 1025 (574 to 1647) | 84.67 (47.39 to 136.02) | -0.784 (-0.791 to -0.777) |
| Oman | 221676 (179134 to 265462) | 65389.96 (52840.86 to 78305.90) | 334535 (261681 to 433762) | 32943.24 (25768.98 to 42714.55) | -2.187 (-2.208 to -2.17) | 3189 (1836 to 5253) | 940.56 (541.45 to 1549.64) | 5725 (3461 to 9277) | 563.73 (340.82 to 913.55) | -1.641 (-1.659 to -1.626) |
| Pakistan | 14593913 (12793175 to 16560350) | 61695.77 (54083.15 to 70008.88) | 34315406 (30292581 to 38991067) | 56650.15 (50009.01 to 64369.05) | -0.279 (-0.284 to -0.273) | 561327 (343281 to 813660) | 2373.01 (1451.22 to 3439.75) | 1100807 (701795 to 1617910) | 1817.28 (1158.57 to 2670.95) | -0.863 (-0.868 to -0.859) |
| Palau | 1709 (1361 to 2157) | 41695.00 (33192.11 to 52616.71) | 1398 (1127 to 1779) | 37327.08 (30086.08 to 47485.88) | -0.362 (-0.367 to -0.356) | 39 (23 to 59) | 952.77 (571.16 to 1435.82) | 33 (20 to 52) | 875.71 (532.48 to 1378.68) | -0.269 (-0.274 to -0.264) |
| Palestine | 149936 (123283 to 184761) | 33917.72 (27888.36 to 41795.69) | 322056 (278691 to 380892) | 24733.56 (21403.21 to 29252.16) | -1.015 (-1.021 to -1.007) | 2866 (1728 to 4463) | 648.25 (390.81 to 1009.58) | 6227 (3673 to 9359) | 478.23 (282.11 to 718.74) | -0.977 (-0.982 to -0.971) |
| Panama | 203098 (150046 to 276478) | 33188.52 (24519.25 to 45179.63) | 251282 (182959 to 361895) | 23508.81 (17116.82 to 33857.24) | -1.109 (-1.134 to -1.093) | 2413 (1271 to 4229) | 394.35 (207.65 to 691.04) | 3108 (1632 to 5387) | 290.79 (152.73 to 503.94) | -0.985 (-1.003 to -0.968) |
| Papua New Guinea | 793156 (659993 to 998874) | 82300.42 (68483.00 to 103646.40) | 1436531 (1117700 to 1827440) | 54908.89 (42722.14 to 69850.74) | -1.312 (-1.338 to -1.285) | 18026 (9580 to 28490) | 1870.41 (994.06 to 2956.24) | 31291 (18909 to 49803) | 1196.03 (722.78 to 1903.62) | -1.429 (-1.452 to -1.407) |
| Paraguay | 326707 (273561 to 402106) | 34750.00 (29097.20 to 42769.80) | 561513 (469270 to 683226) | 29599.78 (24737.25 to 36015.83) | -0.513 (-0.517 to -0.51) | 8615 (5253 to 13407) | 916.35 (558.73 to 1425.99) | 13644 (8342 to 21448) | 719.24 (439.76 to 1130.59) | -0.777 (-0.783 to -0.771) |
| Peru | 2543235 (2042648 to 3138654) | 46816.90 (37601.90 to 57777.63) | 2576397 (2231710 to 3050475) | 26784.88 (23201.43 to 31713.52) | -1.786 (-1.837 to -1.719) | 46299 (27370 to 72770) | 852.28 (503.84 to 1339.59) | 39991 (23994 to 63099) | 415.76 (249.45 to 655.99) | -2.295 (-2.31 to -2.282) |
| Philippines | 6295897 (5476987 to 7207140) | 40568.32 (35291.58 to 46440.02) | 8626251 (7665220 to 9528129) | 29428.12 (26149.60 to 32504.84) | -1.037 (-1.045 to -1.029) | 168004 (101254 to 256176) | 1082.55 (652.44 to 1650.70) | 284263 (173651 to 424001) | 969.75 (592.40 to 1446.46) | -0.348 (-0.354 to -0.342) |
| Poland | 2534083 (2098479 to 3018552) | 26977.13 (22339.81 to 32134.65) | 2017922 (1639473 to 2548276) | 22858.04 (18571.16 to 28865.64) | -0.531 (-0.536 to -0.527) | 66995 (40929 to 101318) | 713.21 (435.72 to 1078.60) | 40588 (24536 to 63044) | 459.76 (277.93 to 714.13) | -1.415 (-1.423 to -1.407) |
| Portugal | 333448 (250127 to 508125) | 13180.15 (9886.74 to 20084.57) | 216303 (174264 to 273524) | 9291.83 (7485.94 to 11749.92) | -1.12 (-1.129 to -1.11) | 4097 (2083 to 7046) | 161.96 (82.35 to 278.51) | 2869 (1513 to 4993) | 123.25 (64.98 to 214.50) | -0.873 (-0.883 to -0.863) |
| Puerto Rico | 298907 (251251 to 378159) | 31198.80 (26224.57 to 39470.81) | 189866 (156096 to 235249) | 25320.36 (20816.79 to 31372.60) | -0.672 (-0.676 to -0.666) | 7031 (4281 to 11165) | 733.84 (446.79 to 1165.34) | 3846 (2305 to 6090) | 512.86 (307.34 to 812.18) | -1.149 (-1.156 to -1.141) |
| Qatar | 34678 (26644 to 44732) | 43858.73 (33697.75 to 56574.81) | 170423 (133216 to 231791) | 30719.16 (24012.45 to 41780.76) | -1.142 (-1.149 to -1.136) | 693 (418 to 1133) | 875.85 (529.20 to 1433.04) | 3135 (1809 to 5145) | 565.11 (326.00 to 927.41) | -1.404 (-1.43 to -1.379) |
| Republic of Korea | 2088496 (1490484 to 3019221) | 16529.47 (11796.49 to 23895.73) | 1064958 (857510 to 1281703) | 9186.41 (7396.95 to 11056.07) | -1.871 (-1.889 to -1.854) | 21507 (10124 to 38845) | 170.22 (80.13 to 307.44) | 15249 (8853 to 24829) | 131.54 (76.37 to 214.18) | -0.807 (-0.829 to -0.786) |
| Republic of Moldova | 342745 (297477 to 402552) | 30453.97 (26431.69 to 35767.92) | 217002 (194237 to 242399) | 24601.02 (22020.13 to 27480.12) | -0.687 (-0.699 to -0.676) | 8085 (4878 to 12023) | 718.35 (433.42 to 1068.29) | 5001 (3134 to 7751) | 566.96 (355.30 to 878.75) | -0.763 (-0.773 to -0.755) |
| Romania | 1496204 (1274558 to 1777697) | 26607.81 (22666.14 to 31613.74) | 929156 (763797 to 1158957) | 22882.29 (18810.01 to 28541.58) | -0.486 (-0.491 to -0.481) | 40817 (25277 to 62998) | 725.88 (449.52 to 1120.32) | 19841 (11926 to 30439) | 488.62 (293.70 to 749.63) | -1.263 (-1.274 to -1.255) |
| Russian Federation | 9135608 (7658197 to 10844258) | 24673.76 (20683.52 to 29288.54) | 7280927 (6165111 to 8718329) | 21481.63 (18189.53 to 25722.54) | -0.446 (-0.458 to -0.434) | 250481 (156399 to 387661) | 676.51 (422.41 to 1047.01) | 178468 (114574 to 275131) | 526.55 (338.04 to 811.75) | -0.807 (-0.82 to -0.79) |
| Rwanda | 465593 (383688 to 581866) | 28696.54 (23648.39 to 35863.00) | 652449 (565627 to 742098) | 18527.33 (16061.86 to 21073.05) | -1.444 (-1.478 to -1.399) | 11638 (7088 to 17823) | 717.29 (436.87 to 1098.51) | 16150 (9848 to 24627) | 458.61 (279.64 to 699.34) | -1.439 (-1.459 to -1.421) |
| Saint Kitts and Nevis | 4308 (3608 to 5314) | 43148.87 (36135.09 to 53225.05) | 5675 (4616 to 7264) | 36346.61 (29566.75 to 46524.69) | -0.552 (-0.557 to -0.546) | 123 (77 to 188) | 1230.87 (774.96 to 1879.89) | 131 (78 to 202) | 838.38 (497.46 to 1290.71) | -1.225 (-1.239 to -1.218) |
| Saint Lucia | 14412 (12174 to 17891) | 42257.72 (35695.03 to 52457.41) | 17280 (14454 to 21357) | 37830.70 (31644.60 to 46757.09) | -0.357 (-0.363 to -0.352) | 434 (261 to 647) | 1271.23 (766.50 to 1897.04) | 439 (275 to 659) | 961.75 (601.09 to 1442.05) | -0.898 (-0.906 to -0.891) |
| Saint Vincent and the Grenadines | 10946 (9202 to 13581) | 41548.41 (34928.96 to 51548.67) | 10898 (9051 to 13817) | 39208.50 (32564.26 to 49709.75) | -0.182 (-0.188 to -0.177) | 325 (195 to 479) | 1233.22 (741.86 to 1817.78) | 280 (172 to 430) | 1005.89 (618.88 to 1548.57) | -0.657 (-0.665 to -0.65) |
| Samoa | 10908 (9783 to 12171) | 29581.37 (26530.58 to 33005.88) | 13347 (11838 to 15163) | 27434.03 (24332.13 to 31166.30) | -0.249 (-0.256 to -0.244) | 387 (254 to 582) | 1050.24 (689.41 to 1579.65) | 454 (279 to 670) | 932.38 (573.42 to 1377.69) | -0.385 (-0.391 to -0.379) |
| San Marino | 508 (402 to 635) | 8158.99 (6463.42 to 10202.05) | 541 (433 to 656) | 7595.13 (6076.29 to 9207.95) | -0.226 (-0.233 to -0.22) | 6 (3 to 11) | 101.55 (55.44 to 172.43) | 7 (4 to 12) | 99.28 (55.75 to 169.08) | -0.068 (-0.081 to -0.057) |
| Sao Tome and Principe | 12641 (9722 to 16278) | 49273.62 (37894.56 to 63449.82) | 24356 (20659 to 29189) | 43421.62 (36830.12 to 52038.08) | -0.424 (-0.465 to -0.375) | 374 (206 to 592) | 1455.96 (801.24 to 2307.80) | 651 (406 to 983) | 1159.89 (724.41 to 1751.87) | -0.789 (-0.825 to -0.752) |
| Saudi Arabia | 1239979 (1120410 to 1386300) | 38023.95 (34357.38 to 42510.90) | 3275813 (2642697 to 4259522) | 32286.17 (26046.23 to 41981.54) | -0.507 (-0.529 to -0.478) | 30048 (18052 to 46936) | 921.43 (553.57 to 1439.30) | 68942 (41030 to 105008) | 679.49 (404.39 to 1034.95) | -0.933 (-0.958 to -0.907) |
| Senegal | 1043363 (916573 to 1191108) | 60635.82 (53267.30 to 69222.14) | 2650374 (2281158 to 3072659) | 67940.94 (58476.28 to 78765.99) | 0.368 (0.356 to 0.381) | 46086 (29398 to 65903) | 2678.34 (1708.49 to 3829.99) | 75729 (47505 to 113583) | 1941.28 (1217.77 to 2911.63) | -1.038 (-1.052 to -1.022) |
| Serbia | 586298 (506371 to 685651) | 25109.94 (21686.82 to 29365.03) | 444719 (367642 to 541022) | 21877.81 (18086.01 to 26615.38) | -0.441 (-0.447 to -0.436) | 16029 (9700 to 24144) | 686.49 (415.45 to 1034.05) | 9565 (5549 to 14922) | 470.52 (273.00 to 734.08) | -1.218 (-1.229 to -1.208) |
| Seychelles | 6981 (5740 to 8990) | 38575.78 (31719.21 to 49676.78) | 8476 (6780 to 10891) | 34964.83 (27968.40 to 44930.22) | -0.318 (-0.324 to -0.313) | 175 (109 to 266) | 967.19 (600.68 to 1467.14) | 189 (120 to 292) | 780.21 (496.83 to 1206.17) | -0.689 (-0.695 to -0.684) |
| Sierra Leone | 490110 (406573 to 596888) | 48815.61 (40495.29 to 59450.84) | 1004263 (845669 to 1191810) | 44158.68 (37185.12 to 52405.38) | -0.322 (-0.335 to -0.308) | 15889 (10010 to 23352) | 1582.56 (996.97 to 2325.86) | 31183 (19335 to 46150) | 1371.14 (850.20 to 2029.27) | -0.466 (-0.478 to -0.453) |
| Singapore | 359865 (195698 to 529324) | 38503.47 (20938.48 to 56634.58) | 264900 (119055 to 506311) | 18210.41 (8184.40 to 34806.08) | -2.399 (-2.418 to -2.382) | 2984 (1180 to 6390) | 319.28 (126.21 to 683.71) | 1956 (523 to 4950) | 134.43 (35.98 to 340.27) | -2.742 (-2.767 to -2.725) |
| Slovakia | 309140 (260850 to 361796) | 23304.89 (19664.52 to 27274.44) | 265192 (218242 to 323189) | 21009.90 (17290.27 to 25604.75) | -0.331 (-0.338 to -0.327) | 8188 (4979 to 13280) | 617.23 (375.37 to 1001.16) | 5573 (3119 to 8962) | 441.49 (247.08 to 710.03) | -1.083 (-1.095 to -1.072) |
| Slovenia | 103122 (87458 to 121969) | 20724.35 (17576.45 to 24512.03) | 73280 (61467 to 88725) | 17422.24 (14613.56 to 21094.15) | -0.561 (-0.565 to -0.557) | 2638 (1663 to 4096) | 530.09 (334.23 to 823.20) | 1525 (922 to 2460) | 362.66 (219.26 to 584.91) | -1.232 (-1.246 to -1.22) |
| Solomon Islands | 39422 (31019 to 51883) | 52972.88 (41681.41 to 69715.99) | 80089 (64680 to 99688) | 46713.98 (37726.35 to 58145.73) | -0.414 (-0.424 to -0.402) | 1076 (620 to 1718) | 1445.77 (832.88 to 2308.12) | 2114 (1318 to 3201) | 1232.80 (768.52 to 1866.91) | -0.514 (-0.528 to -0.499) |
| Somalia | 1292133 (1159472 to 1453972) | 76290.98 (68458.36 to 85846.39) | 2666852 (2066719 to 3295674) | 55140.90 (42732.32 to 68142.67) | -1.051 (-1.067 to -1.038) | 40062 (22635 to 62431) | 2365.34 (1336.43 to 3686.12) | 50536 (30435 to 77867) | 1044.90 (629.29 to 1610.00) | -2.566 (-2.611 to -2.523) |
| South Africa | 3898865 (3301260 to 4639914) | 40388.76 (34198.11 to 48065.36) | 5501768 (4984951 to 6151632) | 35523.42 (32186.47 to 39719.41) | -0.414 (-0.42 to -0.407) | 119748 (74569 to 180148) | 1240.48 (772.47 to 1866.18) | 171693 (110981 to 251961) | 1108.58 (716.57 to 1626.84) | -0.364 (-0.374 to -0.354) |
| South Sudan | 787291 (574821 to 1016174) | 60794.16 (44387.33 to 78468.36) | 1004005 (784723 to 1336396) | 43245.28 (33800.19 to 57562.31) | -1.104 (-1.117 to -1.093) | 15636 (8733 to 24551) | 1207.41 (674.36 to 1895.85) | 21768 (12666 to 33629) | 937.60 (545.56 to 1448.48) | -0.817 (-0.827 to -0.808) |
| Spain | 1012870 (782907 to 1451334) | 10517.08 (8129.27 to 15069.85) | 794101 (638879 to 984873) | 8018.63 (6451.23 to 9945.00) | -0.864 (-0.877 to -0.853) | 11665 (5650 to 20504) | 121.12 (58.67 to 212.90) | 10189 (5469 to 17282) | 102.88 (55.23 to 174.51) | -0.523 (-0.533 to -0.512) |
| Sri Lanka | 2978367 (2507313 to 3464613) | 64876.11 (54615.41 to 75467.73) | 2049000 (1707876 to 2532859) | 36284.02 (30243.33 to 44852.27) | -1.873 (-1.893 to -1.851) | 65079 (39664 to 102722) | 1417.59 (863.98 to 2237.53) | 51020 (31432 to 78342) | 903.46 (556.61 to 1387.28) | -1.443 (-1.453 to -1.434) |
| Sudan | 2599975 (2093485 to 3273830) | 55532.74 (44714.65 to 69925.59) | 5000938 (4044733 to 6436916) | 44299.41 (35829.14 to 57019.62) | -0.703 (-0.726 to -0.677) | 70020 (43489 to 106716) | 1495.55 (928.88 to 2279.34) | 126214 (77585 to 191520) | 1118.03 (687.26 to 1696.52) | -0.897 (-0.924 to -0.862) |
| Suriname | 42245 (35113 to 51503) | 43441.35 (36107.72 to 52961.47) | 59608 (49295 to 73151) | 41073.28 (33967.38 to 50405.23) | -0.18 (-0.183 to -0.176) | 1265 (786 to 1958) | 1300.58 (808.30 to 2013.30) | 1534 (938 to 2329) | 1057.19 (646.63 to 1604.98) | -0.669 (-0.675 to -0.664) |
| Sweden | 191249 (148114 to 268860) | 9316.61 (7215.29 to 13097.40) | 165280 (127785 to 238096) | 7523.11 (5816.46 to 10837.52) | -0.685 (-0.692 to -0.679) | 2395 (1211 to 4156) | 116.66 (59.01 to 202.46) | 2076 (1129 to 3605) | 94.50 (51.40 to 164.11) | -0.672 (-0.68 to -0.665) |
| Switzerland | 255812 (138276 to 476830) | 14532.73 (7855.50 to 27088.85) | 246311 (135500 to 486689) | 12588.96 (6925.41 to 24874.73) | -0.438 (-0.472 to -0.405) | 1643 (468 to 3978) | 93.36 (26.56 to 226.00) | 1750 (553 to 4111) | 89.46 (28.28 to 210.10) | -0.123 (-0.169 to -0.077) |
| Syrian Arab Republic | 1326879 (1058493 to 1670507) | 48120.06 (38386.88 to 60581.94) | 1386722 (1136508 to 1734256) | 35644.22 (29212.73 to 44577.20) | -0.959 (-0.97 to -0.948) | 30924 (19264 to 47331) | 1121.48 (698.61 to 1716.49) | 33067 (19809 to 50664) | 849.95 (509.18 to 1302.26) | -0.887 (-0.904 to -0.872) |
| Taiwan (Province of China) | 1197812 (999701 to 1485819) | 21769.71 (18169.12 to 27004.11) | 942815 (794299 to 1151291) | 16783.10 (14139.36 to 20494.19) | -0.834 (-0.84 to -0.829) | 25866 (15625 to 42634) | 470.11 (283.97 to 774.85) | 19257 (11480 to 31480) | 342.80 (204.36 to 560.38) | -1.017 (-1.026 to -1.009) |
| Tajikistan | 437017 (387307 to 501328) | 36249.03 (32125.77 to 41583.34) | 1024571 (922727 to 1160283) | 40315.29 (36307.89 to 45655.35) | 0.361 (0.334 to 0.382) | 15708 (9880 to 23906) | 1302.89 (819.52 to 1982.91) | 29618 (18541 to 42924) | 1165.43 (729.55 to 1688.98) | -0.355 (-0.364 to -0.347) |
| Thailand | 4429512 (4044184 to 4825598) | 27701.46 (25291.68 to 30178.52) | 3754923 (3270961 to 4270321) | 23034.32 (20065.48 to 26195.99) | -0.592 (-0.602 to -0.584) | 155034 (99800 to 233519) | 969.56 (624.13 to 1460.39) | 116184 (73346 to 178548) | 712.73 (449.93 to 1095.29) | -0.985 (-0.999 to -0.973) |
| Timor-Leste | 69938 (50977 to 87598) | 37531.01 (27355.52 to 47007.44) | 110678 (91652 to 138976) | 31968.38 (26472.73 to 40141.80) | -0.525 (-0.55 to -0.504) | 1772 (995 to 2809) | 951.02 (533.75 to 1507.24) | 1995 (1236 to 3188) | 576.17 (356.89 to 920.79) | -1.629 (-1.656 to -1.605) |
| Togo | 540890 (444972 to 640387) | 63392.63 (52150.98 to 75053.81) | 1482356 (1277551 to 1705276) | 68744.88 (59246.98 to 79082.90) | 0.265 (0.256 to 0.274) | 15362 (9128 to 24378) | 1800.39 (1069.78 to 2857.15) | 35764 (21823 to 54512) | 1658.57 (1012.04 to 2528.02) | -0.259 (-0.273 to -0.245) |
| Tokelau | 161 (129 to 207) | 45581.75 (36554.85 to 58540.39) | 123 (99 to 153) | 38127.45 (30938.25 to 47518.93) | -0.581 (-0.591 to -0.571) | 4 (2 to 6) | 1122.89 (703.61 to 1766.95) | 3 (2 to 4) | 895.11 (569.67 to 1331.67) | -0.731 (-0.74 to -0.722) |
| Tonga | 9040 (7271 to 11356) | 40412.04 (32502.36 to 50765.99) | 9143 (7692 to 11552) | 36161.15 (30422.31 to 45688.33) | -0.36 (-0.363 to -0.356) | 223 (138 to 343) | 994.92 (618.79 to 1534.14) | 224 (133 to 345) | 885.50 (526.49 to 1362.70) | -0.372 (-0.379 to -0.366) |
| Trinidad and Tobago | 126428 (107714 to 155178) | 41099.05 (35015.44 to 50445.07) | 124681 (101850 to 151609) | 36685.41 (29967.88 to 44608.66) | -0.365 (-0.37 to -0.36) | 3650 (2196 to 5550) | 1186.55 (713.75 to 1804.17) | 2894 (1729 to 4533) | 851.45 (508.67 to 1333.75) | -1.07 (-1.08 to -1.06) |
| Tunisia | 638798 (573184 to 707344) | 31243.05 (28033.93 to 34595.61) | 824654 (706315 to 988366) | 26877.87 (23020.88 to 32213.72) | -0.486 (-0.49 to -0.482) | 18342 (11568 to 26920) | 897.09 (565.78 to 1316.62) | 23274 (14963 to 36101) | 758.56 (487.70 to 1176.63) | -0.538 (-0.543 to -0.533) |
| Turkmenistan | 337440 (295999 to 400527) | 38178.18 (33489.58 to 45315.97) | 417459 (359067 to 497392) | 33221.10 (28574.31 to 39582.15) | -1.141 (-1.151 to -1.132) | 11807 (7327 to 17377) | 1335.82 (829.04 to 1966.05) | 12631 (7634 to 19406) | 1005.18 (607.47 to 1544.30) | -1.256 (-1.264 to -1.247) |
| Tuvalu | 1225 (1004 to 1546) | 50330.70 (41236.82 to 63524.90) | 1218 (972 to 1560) | 41926.11 (33457.38 to 53697.61) | -0.45 (-0.454 to -0.445) | 32 (19 to 49) | 1298.38 (791.22 to 1994.10) | 29 (18 to 45) | 1015.27 (622.68 to 1533.02) | -0.923 (-0.934 to -0.913) |
| Turkey | 6223366 (5198135 to 7669920) | 43690.52 (36492.98 to 53845.91) | 6626127 (5477394 to 8185097) | 30603.46 (25297.92 to 37803.72) | -0.59 (-0.594 to -0.585) | 140445 (83317 to 217116) | 985.98 (584.92 to 1524.24) | 144189 (84356 to 231438) | 665.95 (389.61 to 1068.92) | -0.794 (-0.8 to -0.788) |
| Uganda | 1550893 (1299487 to 1879266) | 40303.83 (33770.42 to 48837.42) | 3304873 (2830671 to 3907146) | 31735.82 (27182.19 to 37519.29) | -0.791 (-0.83 to -0.752) | 42094 (25900 to 65591) | 1093.92 (673.07 to 1704.54) | 74807 (46276 to 118107) | 718.35 (444.38 to 1134.15) | -1.362 (-1.398 to -1.326) |
| Ukraine | 2459031 (2180370 to 2743524) | 19425.98 (17224.60 to 21673.44) | 1860356 (1610761 to 2106545) | 18409.79 (15939.84 to 20846.03) | -0.17 (-0.185 to -0.158) | 87540 (55476 to 128438) | 691.56 (438.25 to 1014.64) | 64833 (41545 to 97130) | 641.58 (411.13 to 961.18) | -0.241 (-0.256 to -0.226) |
| United Arab Emirates | 238041 (194945 to 283321) | 69550.25 (56958.46 to 82779.92) | 869426 (640676 to 1140271) | 50431.13 (37162.46 to 66141.55) | -1.023 (-1.037 to -1.008) | 3001 (1691 to 5008) | 876.94 (494.10 to 1463.27) | 13635 (7750 to 22027) | 790.91 (449.55 to 1277.65) | -0.312 (-0.337 to -0.291) |
| United Kingdom | 1369068 (1123116 to 1711007) | 9647.07 (7913.98 to 12056.53) | 1245432 (995992 to 1564653) | 8119.10 (6492.97 to 10200.13) | -0.551 (-0.563 to -0.541) | 19985 (11768 to 32731) | 140.82 (82.92 to 230.64) | 18165 (10343 to 29117) | 118.42 (67.43 to 189.82) | -0.555 (-0.567 to -0.545) |
| United Republic of Tanzania | 4602312 (4397512 to 4831595) | 76702.79 (73289.57 to 80524.06) | 7542155 (6201756 to 9268864) | 50756.56 (41736.06 to 62376.82) | -1.339 (-1.379 to -1.311) | 231207 (157016 to 331597) | 3853.33 (2616.85 to 5526.44) | 195906 (120659 to 302277) | 1318.39 (812.00 to 2034.24) | -3.41 (-3.443 to -3.375) |
| United States of America | 8134091 (7287207 to 9055825) | 12141.39 (10877.28 to 13517.22) | 8996066 (7731620 to 10732268) | 11881.62 (10211.59 to 14174.72) | -0.092 (-0.13 to -0.06) | 112976 (66636 to 174072) | 168.63 (99.46 to 259.83) | 160235 (95850 to 253080) | 211.63 (126.59 to 334.26) | 0.755 (0.719 to 0.782) |
| United States Virgin Islands | 9886 (8242 to 12039) | 34395.82 (28676.19 to 41885.22) | 5386 (4385 to 6870) | 31312.15 (25490.61 to 39934.41) | -0.3 (-0.307 to -0.293) | 233 (142 to 357) | 809.94 (494.97 to 1242.43) | 111 (67 to 175) | 642.39 (392.02 to 1017.48) | -0.758 (-0.78 to -0.739) |
| Uruguay | 141111 (69406 to 267930) | 18789.43 (9241.62 to 35675.70) | 103523 (62948 to 204587) | 12437.02 (7562.44 to 24578.74) | -1.323 (-1.339 to -1.308) | 1407 (528 to 3122) | 187.36 (70.33 to 415.69) | 1032 (395 to 2145) | 123.98 (47.42 to 257.66) | -1.326 (-1.341 to -1.313) |
| Uzbekistan | 2929537 (2629756 to 3300934) | 59802.19 (53682.60 to 67383.69) | 4699624 (4056697 to 5685918) | 52812.79 (45587.79 to 63896.43) | -0.396 (-0.402 to -0.391) | 101505 (67206 to 145132) | 2072.07 (1371.91 to 2962.65) | 157761 (103134 to 234518) | 1772.86 (1158.99 to 2635.43) | -0.509 (-0.515 to -0.501) |
| Vanuatu | 18031 (15838 to 20802) | 51425.94 (45172.84 to 59330.82) | 40838 (31930 to 51571) | 52078.82 (40718.24 to 65766.05) | 0.038 (0.01 to 0.056) | 395 (234 to 608) | 1127.62 (668.72 to 1734.12) | 920 (547 to 1391) | 1173.71 (697.49 to 1773.27) | 0.128 (0.113 to 0.144) |
| Venezuela (Bolivarian Republic of) | 1468123 (893569 to 2062647) | 30329.89 (18460.20 to 42612.16) | 1379407 (1047687 to 2034232) | 20096.98 (15264.05 to 29637.31) | -1.311 (-1.331 to -1.29) | 20454 (10482 to 34686) | 422.56 (216.55 to 716.57) | 22264 (12229 to 38467) | 324.37 (178.17 to 560.44) | -0.851 (-0.871 to -0.829) |
| Viet Nam | 5785553 (5172724 to 6414329) | 33904.22 (30312.95 to 37588.94) | 6340294 (5610652 to 7066055) | 24823.38 (21966.71 to 27664.87) | -1.006 (-1.013 to -0.999) | 178467 (105858 to 264349) | 1045.85 (620.34 to 1549.13) | 201727 (128023 to 303166) | 789.80 (501.23 to 1186.95) | -0.902 (-0.91 to -0.896) |
| Yemen | 1558292 (1407059 to 1742981) | 56889.49 (51368.36 to 63632.04) | 5225261 (4787439 to 5712294) | 62535.63 (57295.80 to 68364.41) | 0.31 (0.304 to 0.315) | 89629 (60950 to 125766) | 3272.15 (2225.15 to 4591.40) | 255532 (171719 to 354469) | 3058.19 (2055.13 to 4242.27) | -0.219 (-0.222 to -0.217) |
| Zambia | 823825 (746686 to 927698) | 44795.89 (40601.46 to 50444.09) | 2023274 (1814312 to 2306299) | 41132.90 (36884.73 to 46886.75) | -0.275 (-0.281 to -0.27) | 38624 (25138 to 55182) | 2100.19 (1366.91 to 3000.56) | 60536 (38534 to 91286) | 1230.68 (783.40 to 1855.83) | -1.729 (-1.75 to -1.71) |
| Zimbabwe | 1192307 (947699 to 1478497) | 49587.86 (39414.62 to 61490.43) | 1432056 (1249919 to 1711800) | 35266.82 (30781.38 to 42155.98) | -1.096 (-1.116 to -1.074) | 26658 (16591 to 40979) | 1108.69 (690.01 to 1704.29) | 40779 (25250 to 61309) | 1004.25 (621.83 to 1509.84) | -0.318 (-0.334 to -0.303) |

AAPC, average annual percentage change; ASPR, age-standardized prevalence rate; CI, confidence interval; UI, uncertainty interval; WRA, women of reproductive age; YLDs, years lost due to disability.

Supplementary Table 2 The predictions of prevalence and YLDs of anemia among WRA from 2022 to 2030.

| Year | Prevalence | | YLDs | |
| --- | --- | --- | --- | --- |
|  | cases (95% UI) | rate per 100,000 (95% UI) | cases (95% UI) | rate per 100,000 (95% UI) |
| 2022 | 673873090 (664084900 to 664084900) | 33747.42 (33257.23 to 34237.6) | 18450860 (18219355 to 18219355) | 924.02 (912.43 to 935.6) |
| 2023 | 678185908 (665910614 to 665910614) | 33743.95 (33133.19 to 34354.72) | 18501576 (18216039 to 18216039) | 920.57 (906.37 to 934.77) |
| 2024 | 682502493 (667839761 to 667839761) | 33738.25 (33013.43 to 34463.07) | 18552456 (18213144 to 18213144) | 917.11 (900.34 to 933.87) |
| 2025 | 686786682 (669678504 to 669678504) | 33730.54 (32890.3 to 34570.78) | 18602982 (18206385 to 18206385) | 913.66 (894.18 to 933.13) |
| 2026 | 690976405 (671231987 to 671231987) | 33719.84 (32756.31 to 34683.37) | 18649963 (18189026 to 18189026) | 910.12 (887.63 to 932.61) |
| 2027 | 694999635 (672324085 to 672324085) | 33704.81 (32605.14 to 34804.48) | 18690395 (18155328 to 18155328) | 906.41 (880.47 to 932.36) |
| 2028 | 698936648 (673021808 to 673021808) | 33686.55 (32437.54 to 34935.56) | 18727225 (18108517 to 18108517) | 902.59 (872.78 to 932.41) |
| 2029 | 702821749 (673318771 to 673318771) | 33667.22 (32253.94 to 35080.49) | 18762870 (18050294 to 18050294) | 898.8 (864.66 to 932.93) |
| 2030 | 706546234 (673052908 to 673052908) | 33648.76 (32053.67 to 35243.86) | 18794879 (17977007 to 17977007) | 895.09 (856.14 to 934.04) |

UI, uncertainty interval; WRA, women of reproductive age; YLDs, years lost due to disability.

Supplementary Table 3 The predictions of prevalence and YLDs of mild anemia among WRA from 2022 to 2030.

| Year | Prevalence | | YLDs | |
| --- | --- | --- | --- | --- |
|  | cases (95% UI) | rate per 100,000 (95% UI) | cases (95% UI) | rate per 100,000 (95% UI) |
| 2022 | 384014982 (376922212 to 376922212) | 19231.39 (18876.19 to 19586.59) | 1409545 (1383110 to 1383110) | 70.59 (69.27 to 71.91) |
| 2023 | 387410193 (378247471 to 378247471) | 19276.06 (18820.16 to 19731.96) | 1422107 (1388065 to 1388065) | 70.76 (69.07 to 72.45) |
| 2024 | 390827233 (379764654 to 379764654) | 19319.82 (18772.97 to 19866.68) | 1434773 (1393729 to 1393729) | 70.93 (68.9 to 72.95) |
| 2025 | 394241419 (381319384 to 381319384) | 19362.6 (18727.95 to 19997.24) | 1447452 (1399542 to 1399542) | 71.09 (68.74 to 73.44) |
| 2026 | 397632253 (382803618 to 382803618) | 19404.56 (18680.93 to 20128.2) | 1460063 (1405102 to 1405102) | 71.25 (68.57 to 73.93) |
| 2027 | 400971113 (384123255 to 384123255) | 19445.56 (18628.5 to 20262.61) | 1472502 (1410068 to 1410068) | 71.41 (68.39 to 74.44) |
| 2028 | 404293439 (385291980 to 385291980) | 19485.67 (18569.86 to 20401.48) | 1484903 (1414493 to 1414493) | 71.57 (68.18 to 74.96) |
| 2029 | 407603603 (386283074 to 386283074) | 19525.4 (18504.09 to 20546.72) | 1497284 (1418277 to 1418277) | 71.72 (67.94 to 75.51) |
| 2030 | 410833258 (386993500 to 386993500) | 19565.64 (18430.29 to 20700.99) | 1509391 (1421038 to 1421038) | 71.88 (67.68 to 76.09) |

UI, uncertainty interval; WRA, women of reproductive age; YLDs, years lost due to disability.

Supplementary Table 4 The predictions of prevalence and YLDs of moderate anemia among WRA from 2022 to 2030.

| Year | Prevalence | | YLDs | |
| --- | --- | --- | --- | --- |
|  | cases (95% UI) | rate per 100,000 (95% UI) | cases (95% UI) | rate per 100,000 (95% UI) |
| 2022 | 266672830 (263172728 to 263172728) | 13354.92 (13179.64 to 13530.19) | 13609351 (13430544 to 13430544) | 681.55 (672.61 to 690.5) |
| 2023 | 267798771 (263535841 to 263535841) | 13324.65 (13112.55 to 13536.75) | 13667713 (13449973 to 13449973) | 680.05 (669.23 to 690.88) |
| 2024 | 268914161 (263876052 to 263876052) | 13293.27 (13044.23 to 13542.32) | 13725585 (13468252 to 13468252) | 678.5 (665.78 to 691.21) |
| 2025 | 270011361 (264135233 to 264135233) | 13261.22 (12972.62 to 13549.81) | 13782587 (13482430 to 13482430) | 676.91 (662.17 to 691.65) |
| 2026 | 271054261 (264228241 to 264228241) | 13227.52 (12894.42 to 13560.63) | 13836861 (13488156 to 13488156) | 675.24 (658.23 to 692.26) |
| 2027 | 272007132 (264081844 to 264081844) | 13191.3 (12806.96 to 13575.64) | 13886614 (13481721 to 13481721) | 673.45 (653.81 to 693.08) |
| 2028 | 272899498 (263729608 to 263729608) | 13152.9 (12710.94 to 13594.85) | 13933325 (13464806 to 13464806) | 671.54 (648.96 to 694.12) |
| 2029 | 273759787 (263189422 to 263189422) | 13113.89 (12607.55 to 13620.24) | 13978424 (13438301 to 13438301) | 669.61 (643.74 to 695.48) |
| 2030 | 274553614 (262409509 to 262409509) | 13075.42 (12497.07 to 13653.77) | 14020157 (13399564 to 13399564) | 667.7 (638.15 to 697.25) |

UI, uncertainty interval; WRA, women of reproductive age; YLDs, years lost due to disability.

Supplementary Table 5 The predictions of prevalence and YLDs of severe anemia among WRA from 2022 to 2030.

| Year | Prevalence | | YLDs | |
| --- | --- | --- | --- | --- |
|  | cases (95% UI) | rate per 100,000 (95% UI) | cases (95% UI) | rate per 100,000 (95% UI) |
| 2022 | 23575076 (23195544 to 23195544) | 1180.63 (1161.63 to 1199.63) | 3442244 (3387003 to 3387003) | 172.39 (169.63 to 175.15) |
| 2023 | 23463631 (22982875 to 22982875) | 1167.46 (1143.55 to 1191.37) | 3426249 (3356446 to 3356446) | 170.48 (167.01 to 173.95) |
| 2024 | 23356718 (22783534 to 22783534) | 1154.6 (1126.27 to 1182.92) | 3410906 (3327764 to 3327764) | 168.61 (164.51 to 172.72) |
| 2025 | 23253454 (22589261 to 22589261) | 1142.06 (1109.44 to 1174.67) | 3396087 (3299781 to 3299781) | 166.79 (162.07 to 171.52) |
| 2026 | 23147860 (22388605 to 22388605) | 1129.62 (1092.57 to 1166.67) | 3380930 (3270849 to 3270849) | 164.99 (159.62 to 170.36) |
| 2027 | 23035228 (22172672 to 22172672) | 1117.12 (1075.29 to 1158.95) | 3364723 (3239656 to 3239656) | 163.18 (157.11 to 169.24) |
| 2028 | 22921790 (21947152 to 21947152) | 1104.76 (1057.79 to 1151.73) | 3348387 (3207044 to 3207044) | 161.38 (154.57 to 168.19) |
| 2029 | 22811966 (21715021 to 21715021) | 1092.76 (1040.21 to 1145.3) | 3332565 (3173449 to 3173449) | 159.64 (152.02 to 167.26) |
| 2030 | 22702321 (21471107 to 21471107) | 1081.18 (1022.55 to 1139.82) | 3316748 (3138113 to 3138113) | 157.96 (149.45 to 166.46) |

UI, uncertainty interval; WRA, women of reproductive age; YLDs, years lost due to disability.


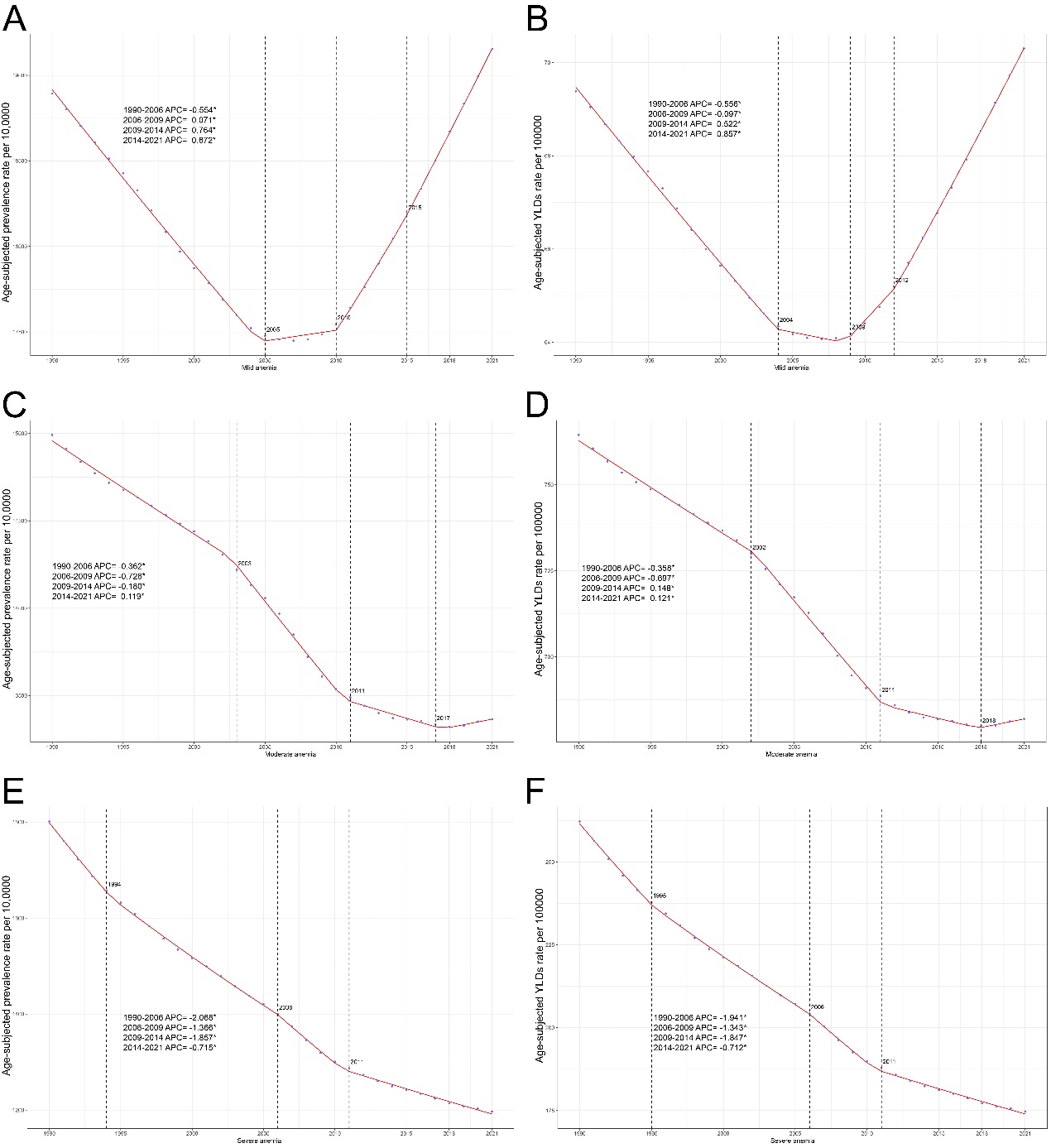


Supplementary Figure 1. Joinpoint regression analysis of mild, moderate and severe anemia among WRA. (A), (C) and (E) Age-standardized prevalence rate per 100,000 from 1990 to 2021, and (B), (D) and (F) Age-standardized YLDs rate per 100,000 from 1990 to 2021. APC, annual percentage change; WRA, women of reproductive age; YLDs, years lost due to disability.


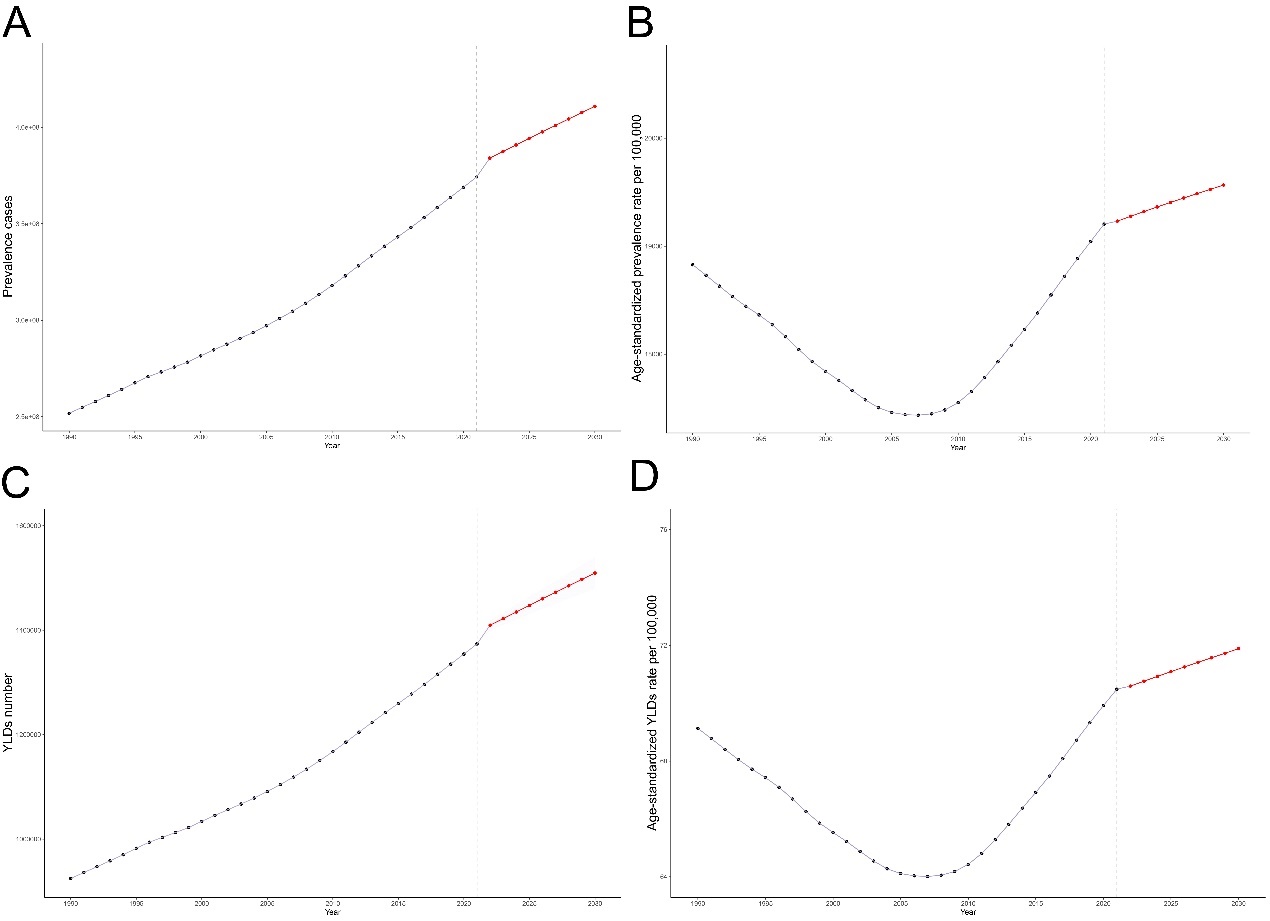


Supplementary Figure 2. (A) Prevalence cases, (B) Age-standardized prevalence rate per 100,000, (C) YLDs number, and (D) Age-standardized YLDs rate per 100,000 of mild anemia among WRA from 1990 to 2021, and their predictions to 2030. The known data from 1990 to 2021 and predicted data from 2022 to 2030 were divided by a grey dashed line. Abbreviations as in Supplementary Figure 1.


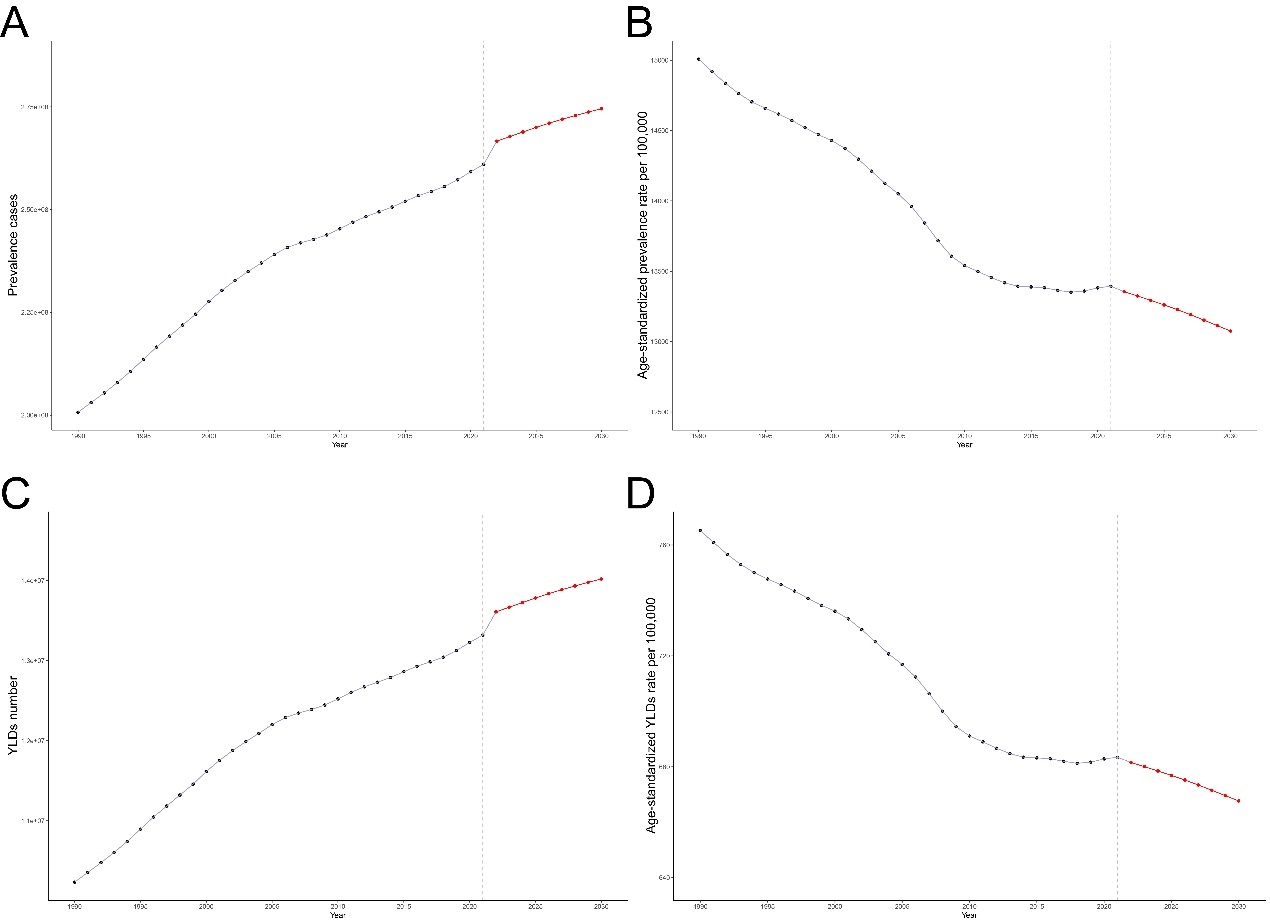


Supplementary Figure 3. (A) Prevalence cases, (B) Age-standardized prevalence rate per 100,000, (C) YLDs number, and (D) Age-standardized YLDs rate per 100,000 of moderate anemia among WRA from 1990 to 2021, and their predictions to 2030. The known data from 1990 to 2021 and predicted data from 2022 to 2030 were divided by a grey dashed line. Abbreviations as in Supplementary Figure 1.


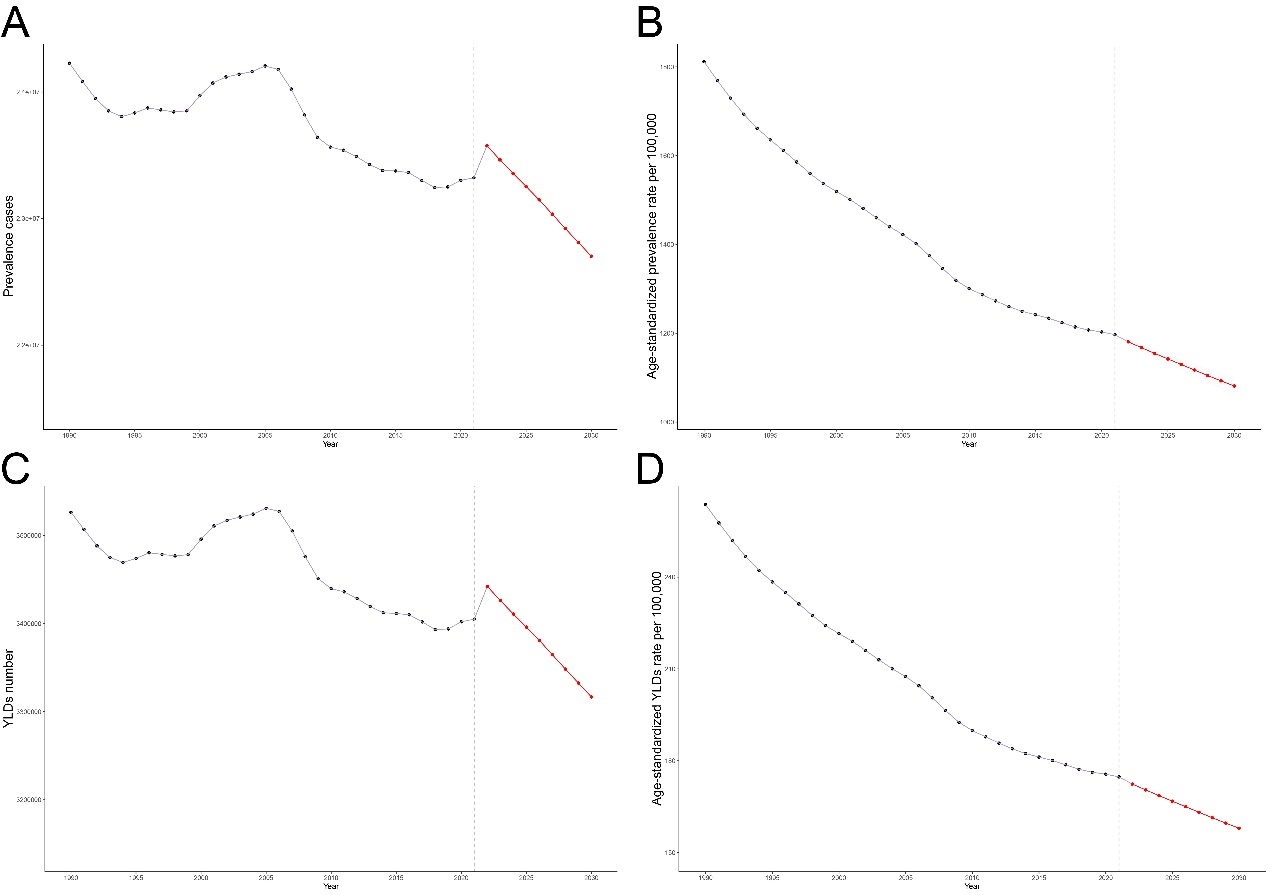


Supplementary Figure 4. (A) Prevalence cases, (B) Age-standardized prevalence rate per 100,000, (C) YLDs number, and (D) Age-standardized YLDs rate per 100,000 of severe anemia among WRA from 1990 to 2021, and their predictions to 2030. The known data from 1990 to 2021 and predicted data from 2022 to 2030 were divided by a grey dashed line. Abbreviations as in Supplementary Figure 1.
